# scSTEM: clustering pseudotime ordered single-cell data

**DOI:** 10.1186/s13059-022-02716-9

**Published:** 2022-07-07

**Authors:** Qi Song, Jingtao Wang, Ziv Bar-Joseph

**Affiliations:** 1 Computational Biology Department, School of Computer Science, Carnegie Mellon University, Pittsburgh, PA 15213 USA; 2 Department of Medicine, Division of Experimental Medicine, McGill University, Montreal, QC Canada; 3 Machine Learning Department, School of Computer Science, Carnegie Mellon University, Pittsburgh, PA 15213 USA

**Keywords:** Single cell, Genomics, Gene clustering, Visualization

## Abstract

We develop scSTEM, single-cell STEM, a method for clustering dynamic profiles of genes in trajectories inferred from pseudotime ordering of single-cell RNA-seq (scRNA-seq) data. scSTEM uses one of several metrics to summarize the expression of genes and assigns a *p*-value to clusters enabling the identification of significant profiles and comparison of profiles across different paths. Application of scSTEM to several scRNA-seq datasets demonstrates its usefulness and ability to improve downstream analysis of biological processes. scSTEM is available at https://github.com/alexQiSong/scSTEM.

**Supplementary Information:**

The online version contains supplementary material available at 10.1186/s13059-022-02716-9.

## Background

Much attention in the analysis of single-cell data has focused on grouping cells to cell types or on modeling trajectories of cell development and differentiation [1, 2]. Much less work in this area has focused on the analysis of genes within identified clusters or for a given path within reconstructed trajectories. Methods that have been developed for these tasks, including scLM [3], and GPseudoClust [4], have mainly focused on clustering genes within cell types and did not utilize dynamic information obtained from time series scRNA-Seq studies or from other trajectory inference methods.

A unique aspect of scRNA-Seq data is the ability to extract detailed dynamics even when using a small number, or a single, time point. Such analysis, which is often termed pseudotime ordering of cells results in a reconstructed trajectory of cells along a (usually small) number of branches and paths. An important question for the resulting trajectory is, What are the sets of expressed and repressed genes that are activated or repressed along a specific branch in the model and how different branches vary in such sets? This information can be very useful in determining the function or type of cells along a certain path [5]. Moreover, comparing such clusters between two paths that split at a certain point in the pseudotime can help explain the differences that led to the separation and in some cases can be used to predict specific interventions that may affect cell fate decisions [6].

**Fig. 1:**
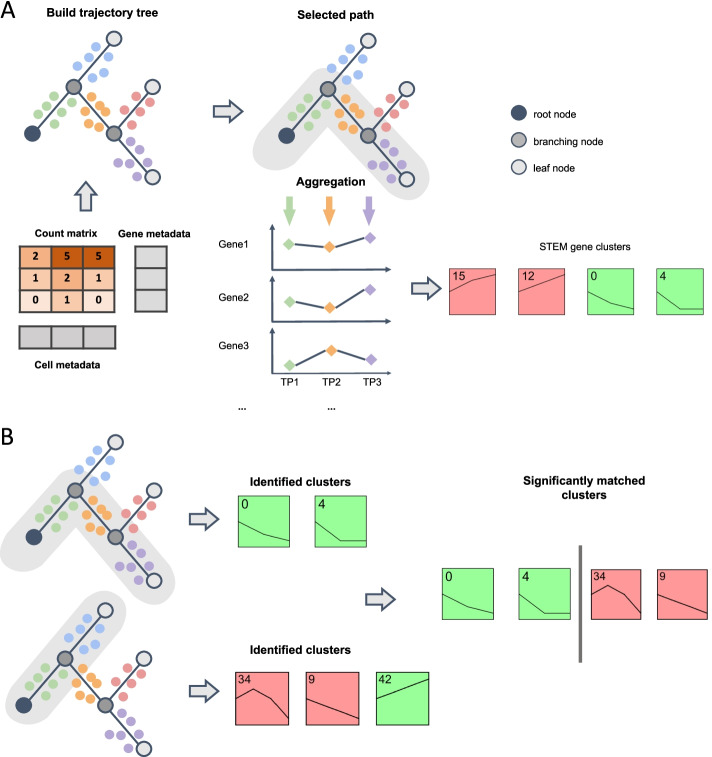
Flowchart of scSTEM pipeline. **A** The flowchart of scSTEM gene clustering. Colored solid circles represent cells mapped to different key segments of a trajectory tree. Aggregation of expressions are performed for each key segment. The resulted matrix (rows as genes and column as key segments) will be used as input for STEM to perform gene clustering. **B** Comparison function enables comparison of clustering results from different trajectory paths. This will identify clusters having similar genes but showing different/similar temporal expression patterns

While clustering of genes along paths is of interest, it is also challenging. As mentioned above, the number of branching points in an inferred trajectory is usually quite small [7, 8]. This means that genes in different paths often share parts of the paths and, assuming a constant rate change along a specific edge in the path, that the actual number of different values we can obtain for a gene along each of the paths is fairly small (Fig. 1). Thus, any clustering methods that would attempt to cluster pseudotime ordered data would have to account for the large overlap and the relatively small number of effective time points.

To provide such method that can be used to cluster pseudotime ordered data, we extended the Short Time-series Expression Miner (STEM) [9] so that it can be applied to scRNA-Seq data. STEM was originally developed for short time series bulk data and employs a unique approach to identify significant clusters. Unlike most methods that are data driven, STEM pre-computes the set of all possible expression profiles. It then assigns genes to the pre-computed expression profiles and groups similar profiles to create clusters. By using a pre-defined set of profiles, STEM can assign significance to each cluster by comparing the enrichment of a profile to an enrichment of the same profile for randomized temporal data. Since STEM needs to account for all possible profiles, such method can only work for short time series datasets [10]. STEM is the most widely used method for clustering time series bulk data, though until now was not able to cluster single-cell data.

Here, we extended STEM and developed scSTEM, which can use trajectory information from single-cell data to cluster genes. To perform such clustering, scSTEM first uses one of several pseudotime inference methods to construct a trajectory for a given scRNA-Seq data. Next, for every gene in every connected component in the analysis scSTEM generates summary time series data using several different approaches for each of the paths. This data is then used as input for STEM and clusters are determined for each path in the trajectory. Users can also compare STEM clusters between two trajectories in the same component to determine what are the differences in genes and biological processes that led to the divergence of these trajectories. We compared scSTEM to several other methods and show that scSTEM produced the best functional relevant clusters and scales well to large single-cell datasets. We have tested scSTEM on a number of datasets with different trajectory inference methods and gene summarization methods. As we show, scSTEM can correctly identify the key functional processes expected to be active along different paths. In addition, comparisons using scSTEM provide biological insights about the activity of different cell types, including clusters distinguishing between very similar cell types such as T cells and NK cells.

## Results

### scSTEM—clustering time series scRNA-Seq data

The overall idea for scSTEM is to cluster genes based on their temporal expression patterns along a given trajectory path. The clustering process starts with building a trajectory tree using expression count matrix, gene metadata, and cell metadata as input files. scSTEM can work with several trajectory inference methods including several popular methods (Monocle 3 [11], Slingshot [7], PAGA [12], etc., see “Methods”). For an input dataset, following trajectory inference a user can select one of the paths using the scSTEM GUI. scSTEM then uses one of the several metrics (“Methods”) to summarize the expression profiles of genes along the selected path. These values, for each gene, are then used as inputs for STEM clustering. By using aggregated gene expressions along each path, scSTEM reduces the impact of noise and dropouts while still taking full advantage of scRNA-Seq data to identify many different trajectories within a time series dataset. The final output from scSTEM for each path includes (1) a table of enriched GO terms for each cluster, (2) a table of gene list and corresponding cluster assignments, and (3) a plot showing temporal gene expression profiles. Each profile represents a small set of genes with similar temporal pattern, and significant profiles are further grouped together to construct larger clusters. Figure 1A illustrates each key step of the scSTEM pipeline. Apart from clustering genes on each individual path, scSTEM also allows for comparison of clustering results between different paths (Fig. 1B). This would match clusters having similar genes that may be expressed differently in different branches. In the following subsections, we will demonstrate application of scSTEM to a number of time series scRNA-seq data sets.

### Identification of functional gene clusters using scSTEM

We applied scSTEM to three scRNA-seq data sets: (1) human fetal immune cells [13], (2) mouse embryonic blood cells [11], (3) mouse embryonic neural crest cells [11]. For each data set, we selected a subset of genes for our analysis. These genes can either be top-ranked cell-type-specific marker genes or highly variable genes. We have found that such filtering is important for trajectory inference and for scSTEM to produce meaningful and compact gene clusters (Additional File 2: Fig. S1). We preprocessed the expression count matrix, cell metadata, and gene metadata as described in “Methods”.

#### Human fetal immune cells [13]

**Fig. 2:**
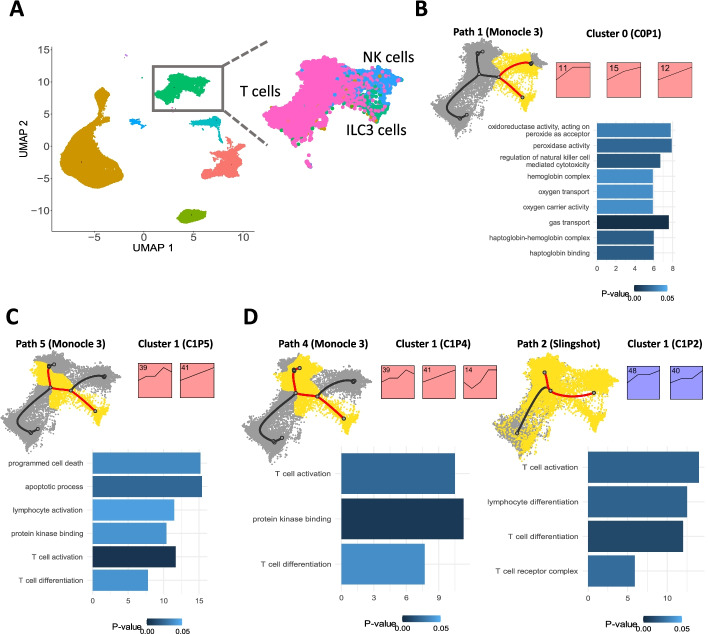
scSTEM results for human fetal immune cells. **A** UMAP visualization of human fetal immune data set. **B** scSTEM results for path 1 (NK cell-related path), with top 10 enriched GO terms (ranked by enrichment fold). **C** Comparison of scSTEM results between using Monocle3 and using Slingshot as trajectory inference method. The top 10 enriched GO terms have shown T cell-related activities. **D** scSTEM results and enriched GO terms for path 5, a T cell-related path. The top 10 enriched GO terms have shown T cell-related activities. Black curves indicate the trajectory tree, and the highlighted red curves are edges along one selected path. Yellow cells are cells mapped to the selected path and grey cells are other remaining cells

In this study, researchers looked at fetus development and profiled cells from several different tissues over time. To focus on a specific biological process, we have analyzed the set of blood cells from this dataset (103,766 cells sampled at 18 time points). Cell type annotations provided in the original publication [13] have identified several immune cell clusters within these blood cells including ILC3 cell, T cell, and NK cell cluster. We next used Monocle 3 and mean expression to perform scSTEM clustering analysis for the immune cell partition for this data set. Monocle 3 identified 7 paths, and scSTEM analysis of these resulted in several significant clusters (between 1 and 5 significant clusters per path, each with 35 ~ 301 gene) (Additional file 1). These clusters successfully captured key properties of these cells and the genes activated during their development. As can be seen in Fig. 2, cluster 0 in path 1 is associated with regulation of NK cell-mediated cytotoxicity (Fig. 2B), while cluster 1 in path 5 and cluster 1 in path 4 are both associated with T cell activation and differentiation (Fig. 2C, D). We have also tested other methods for pseudotime inference with this data and observed similar results. For example, the T cell activation and differentiation cluster and path were also captured by using Slingshot and mean expressions (Fig. 2C). Expressions of genes in significant clusters mostly show an increase in levels as cells transition along the trajectory enabling the identification of those genes that are likely associated with immune cell fates (Fig. 2B–D). Using additional trajectory inference methods and other expression summarization methods leads to similar results as shown in Additional file 2: Fig. S2 and Additional file 2: Fig. S3.

#### Mouse embryonic blood cells

**Fig. 3:**
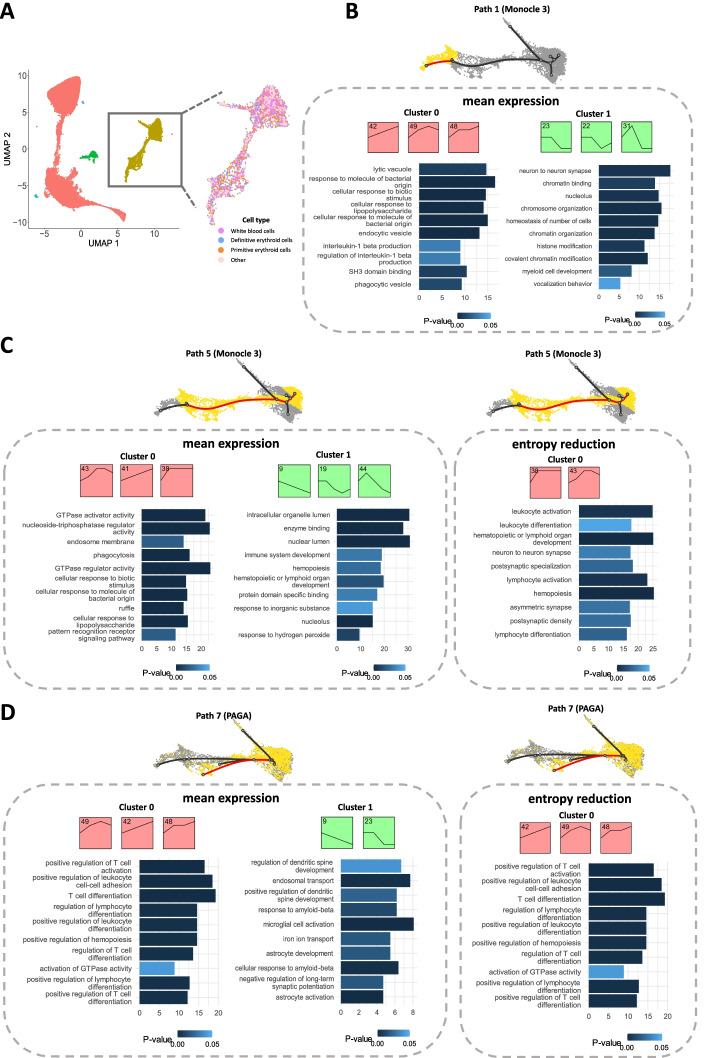
scSTEM results for mouse embryonic blood cells and comparison for different trajectory inference methods and different gene summarization methods. **A** UMAP visualization of mouse embryonic blood cell data set. **B** scSTEM results for path 1, with top 10 enriched GO term (ranked by enrichment fold). **C** scSTEM results and the top 10 enriched GO terms for different trajectory inference methods and different gene summarization methods. Black curves indicate the trajectory tree, and the highlighted red curves are edges along one selected path. Yellow cells are cells mapped to the selected path and grey cells are other remaining cells

This data set investigated gene expressions during early organogenesis of mouse embryo [11]. In this analysis, we applied scSTEM to erythroid and white blood cells (42,262 cells sampled from 5 time points). We used the cell type annotations as given in the original publication [11]. Although there is no clear separation of cell types in the 2D UMAP space, scSTEM was able to identify significant clusters consisting of immune development and immune response-related genes (Fig. 3B, C, interleukin-related terms and phagocytosis-related terms). Using Monocle3 and mean expressions, scSTEM has identified 6 paths each with between 1 and 5 significant clusters, and each path contains 52 ~ 401 genes (Additional file 1). Interestingly, in contrast to the increased expressions of immune response-related clusters, cell development/immune system development-related clusters are characterized by decreased expression levels of genes assigned to these profiles (cluster 1 in Fig. 3B and cluster 1 of path 5 identified by Monocle 3 + mean expressions in Fig. 3C). This is likely the result of fixation of the terminal cell fate as time progresses which leads to repression of developmental programs in these cells. In contrast, as time progresses more and more, fate (immune)-related genes are expressed at higher levels as indicated by the profiles. Therefore, scSTEM can reveal functional gene clusters modulating different biological processes, as well as their temporal expression patterns. For this data set, we also applied PAGA and entropy reduction as additional trajectory inference method and additional gene summarization method and compared them to the Monocle 3 + mean expression results. As illustrated in Fig. 3C, PAGA and Monocle 3 have identified similar paths and clusters, showing increased immune response activity by mean expression and entropy reduction. Results for other trajectory inference methods can be found in Additional file 2: Fig. S4 and Additional file 2: Fig. S5.

#### Mouse embryonic neural crest cells

**Fig. 4:**
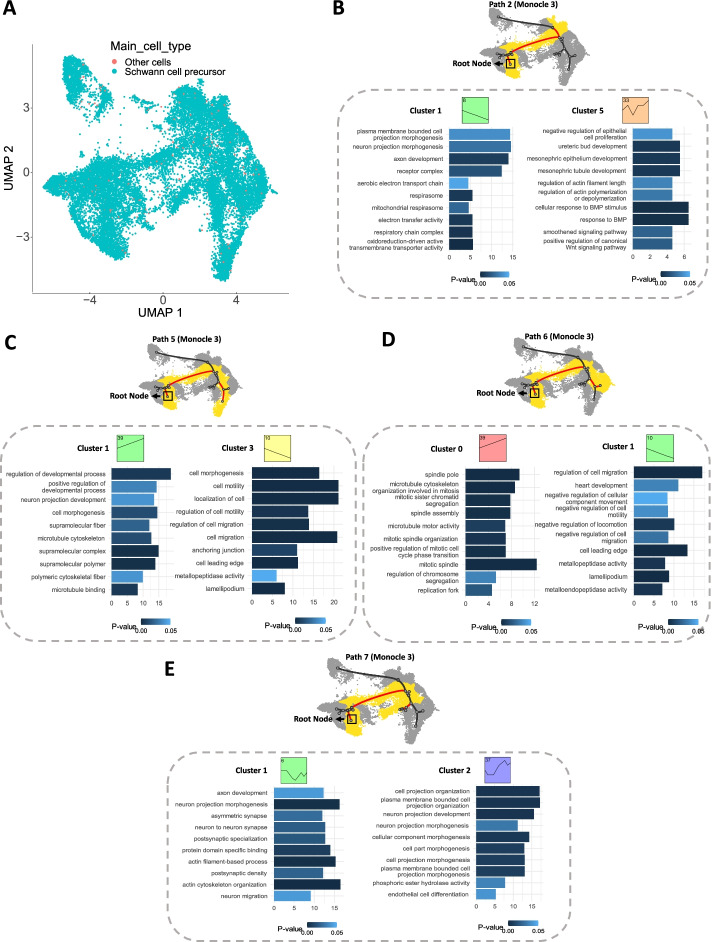
scSTEM results for mouse embryonic neural crest cells. **A** UMAP visualization of mouse embryonic neural crest cell data set. **B–D** scSTEM results and the top 10 enriched GO terms (ranked by enrichment fold) for clusters in different trajectory paths. Black curves indicate the trajectory tree, and the highlighted red curves are edges along one selected path. Yellow cells are cells mapped to the selected path and grey cells are other remaining cells

Finally, we applied scSTEM with Monocle 3 and mean expression to neural crest cells in mouse embryo organogenesis data set (22,283 cells sampled from 5 time points). scSTEM has identified 12 paths with 1 ~ 6 significant clusters (Additional file 1). Several of the significant clusters identified by scSTEM are related to neuron development (Fig. 4B, C, E), and these clusters can be used to identify new genes associated with these functions. Note that these clusters showed diverse expression patterns. For example, cluster 1 in path 2 (Fig. 4B) and cluster 1 in path 7 (Fig. 4E) show decreased expressions for neuron/axon development-related genes, while cluster 1 in path 5 (Fig. 4C) represents increased expressions for neuron/axon development. This suggests the same functional module may drive cells towards different terminal fates through modulation of gene expressions. Results for additional trajectory inference and gene summarization methods can be found in Additional file 2: Fig. S6 and Additional file 2: Fig. S7.

### scSTEM can characterize functional role of unannotated cell clusters

One challenge users may face is to determine the functional role of cell cluster when cell type annotation is not available. Here, we demonstrate that scSTEM is able to characterize the functional role of unannotated cells in the human fetal immune cell data set [13]. We applied scSTEM to the largest unannotated cell cluster and its adjacent cluster in spleen tissue using Monocle 3 + mean expression. In the original publication, the authors did not identify a specific cell type for these cells but marked this cluster based on its highly expressed genes STC2 and TLX1. We therefore used “STC2_TLX1” to label this cell cluster. This cell cluster may represent progression from an unknown cell type towards stromal cell (Additional File 2: Fig. S8 A B). The results showed that scSTEM has identified cluster0 in path3 as significant. This cluster is characterized by a strong decline in expressions, which may signify its role during the cell transition. GO analysis further revealed that cluster 0 is likely to associate with “antigen processing and presentation of endogenous antigen” (corrected *p*-value = 0.004, Additional File 2: Fig. S8 C). The discussions in the supplementary materials of human fetal immune cell data set paper [13] suggested this STC2_TLX1 cell cluster may relate to mesenchymal precursors or stem cells. These cells have been reported to activate antigen presentation [14, 15]. Although the true cell type of STC2_TLX1 cell cluster remains undetermined, scSTEM analysis may help to annotate this unknown set of cells based on the profiles and function of the clusters.

### Comparison of functional gene clusters using scSTEM

In addition to clusters of expressed or repressed genes within specific paths, an important biological question is the identification of differentially activated pathways and program between different conditions, cell types, and developmental stages. For example, an important question in single-cell analysis is determining the reasons for a branching point in which some cells continue to one fate whereas others continue to another. A possible way to address this is to compare the expression of significant clusters between two paths in a trajectory to determine if the same set of genes display significantly different expression patterns following the branching point. scSTEM can be used to identify such differences between two cluster sets by identifying significant intersections in genes assigned to profiles in two different paths (“Methods”).

**Fig. 5:**
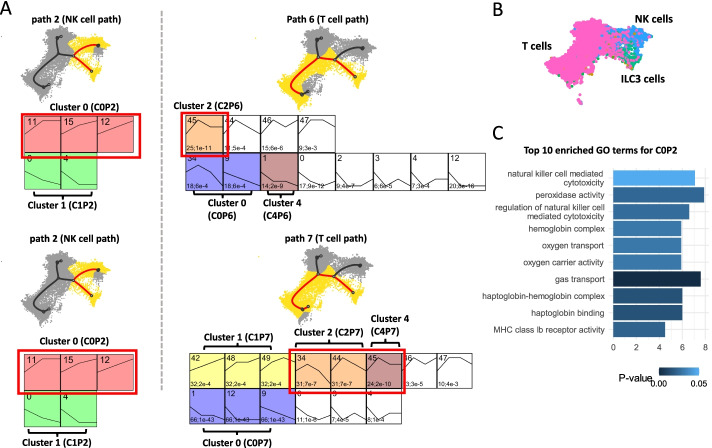
Comparison of functional gene clusters in human fetal immune cell data set. **A** The Comparison for clustering results between NK cell path and T cell paths. Each row on the left side represents one scSTEM cluster from path 2 (NK cell path), which significantly overlapped with each cluster shown on the right side (clusters from T cell path). Highlighted clusters are the ones significantly enriched for NK cell markers in each path. Black curves indicate the trajectory tree, and the highlighted red curves are edges along one selected path. Yellow cells are cells mapped to the selected path and grey cells are other remaining cells. **B** Differentiation of T cells and NK cells. **C** The top 10 enriched GO terms for path 2 (NK cell path, ranked by enrichment fold), cluster 0 (highlighted in **A**)

We performed the comparative analysis using human fetal immune cell data set to compare the clustering results related to the two very similar cell populations, T cells and NK cells. By using Monocle 3 + mean expressions, we have identified clusters with similar genes that showed discrepant expression patterns in different paths. As can be seen Fig. 5A, path 2 is related to progression towards NK cells while path 6 and path 7 are related to progression towards T cells. These two cell types are fairly similar, and identification of the specific program that differs between them is of interest. scSTEM identified that genes assigned to Cluster 0 in path 2 (C0P2, 301 genes) significantly overlap genes assigned to cluster 2 in path 7 (C2P7, 124 genes) and cluster 2 in path 6 (C2P6, 56 genes). See Additional file 3 for genes assigned to the clusters in each path. Genes in C0P2 display elevated expressions (See Additional File 2: Fig. S9 for detailed visualizations of gene profiles and clusters) along NK cell developmental path, and GO enrichment analysis indicates that these genes are significantly associated with NK cell cytotoxicity (Fig. 5C and Additional file 4). In contrast, for the T cell path, the same genes are assigned to C2P7 and C2P6 which display increased expressions at the beginning (shared part of the path) but decreased expressions later when the T cell fate is achieved. Interestingly, many of the genes in C0P2 are known NK markers (14 genes, hypergeometric test, *p*-value < 0.001). These markers include SAMD3, a signal transducer protein that has been reported to have reduced expressions in T cells [16]. GZMM and GZMA were also included in the C0P2 cluster. These are granzymes that mediate targeted cell death through NK cells [17]. GZMM is reported to be primarily expressed in NK cells and has shown reduced expressions in other innate T cells [17]. Although GZMA has been reported to be expressed both in T cells and NK cells, the observed expression patterns may indicate that GZMA has a stronger role in NK cells, which is less characterized in previous studies. Thus, by identifying differential expression patterns for significant clusters, scSTEM was able to zero in on some of the key NK-specific genes and show that their expression indeed differs in T cells.

To further validate the role of the genes, we compared all three clusters to 80 available human NK cell expression markers [18] (https://www.panglaodb.se/markers.html?cell_type=%27NK%20cells%27). We performed hypergeometric test and used all protein coding genes (19,965 genes) in GRCh38 genome [19] as background. The results showed that C0P2 has 14 NK cell markers (*p*-value < 0.001) and C2P7 has 4 NK cell markers (*p*-value < 0.001) and no NK cell markers were found in C2P6. The comparison between C0P2 and C2P7 is consistent with the expectation that NK cell marker genes showed increased expression early in the common developmental path between NK cells and T cells (Fig. 5A, B) but later in the more differentiated NK cell-specific path and T cell-specific path, these genes showed drastically different expression patterns, possibly leading to different cell fates. The above analysis used all protein coding genes as background for hypergeometric test. We have also performed a stricter analysis in which we compared scSTEM significant clusters to the subset of 3000 genes which passed the initial filtering. For this analysis, we still observed a significant enrichment of NK cell markers for C0P2 (*p*-value = 0.012) though the enrichment for C2P7 was not significant (*p*-value = 0.240) when using this reduced background.

### Comparison with previous clustering methods

To further evaluate the clusters identified by scSTEM, we compared scSTEM clustering results to a number of other single-cell gene clustering methods (scHOT [20] and tradeSeq [5]). Since a common workflow for single-cell analysis is to first identify differentially expressed (DE) genes and then perform GO enrichment analysis based on rankings by *p*-values from DE analysis, we have also compared our pipeline to two single-cell DE methods (Monocle 3 and tradeSeq [5]). In this analysis, we focused on the NK cell-related path in human fetal immune cell data set [13] (3000 genes and 4058 cells). Since there is no standard metric for comparing gene clustering results, we used NK gene ratio to evaluate the results (number of genes annotated with natural-killer-cell-related functions divided by cluster size or DE gene number). Results show that scSTEM achieved the best functional relevance, with highest NK gene ratio among all methods (5.98% for scSTEM, 0% for scHOT, 3.39% for Monocle 3, 3.72% for tradeSeq in DE analysis, and 0% for tradeSeq in clustering analysis). See Additional file 2: Fig. S10 A B for complete results. As for computation efficiency, scSTEM is the second-fastest method among all methods (16 s for Monocle3, 60 s for scSTEM, > 10,000 s for tradeSeq and > 40,000 s for scHOT). Additionally, scSTEM only produced two significant clusters, as compared to 66 clusters from tradeSeq and 6 clusters from scHOT, which may make the clustering results easier to interpret. To evaluate whether scSTEM can capture underlying biological signals from DE genes, we generated synthetic scRNA-seq datasets with simulated DE genes. (See Additional File 2: Supplementary Note for results).

## Discussion

Several methods have been developed to reconstruct trajectories using single-cell data. To enable downstream analysis of the resulting trajectories and the clustering of genes along paths in each trajectory, we extended the STEM method and developed scSTEM. scSTEM retains much of the features that made STEM a big success in bulk time series analysis. Specifically, scSTEM is able to assign significance to profiles and to group similar profiles to form clusters. Since the clusters are not data driven, scSTEM also allows the ability to compare clusters between trajectories to infer key differences that may indicate which biological processes or functions leading to the observed branching in the inferred trajectory.

To enable the use of single-cell data in STEM, we combined it with several popular pseudotime inference methods. Users can choose one of these methods to infer trajectories and then perform scSTEM analysis on the resulting paths. scSTEM provides a number of options for users for summarizing genes along an edge in the path. The first is mean expression which works well for cases where the expression of genes along a specific path is expected to be homogeneous (for example, in cases where the data is sampled at a high rate and so there are only small differences between cells on the same path). Rate change may be a useful metric for cases where cells are expected to change along a path in a predictable way (for example during development, some pathways are expected to be steadily increasing whereas others decreasing). Finally, entropy can be used for cases where little is known about the underlying molecular activity in each path.

Once the user selects a trajectory inference and gene summarization method, scSTEM clusters cells along each path. These clusters are easy to interpret and are represented by cluster plots in which the trend of expressions corresponds to the progression of cell fates along each specific trajectory path. Gene clustering of each path is fast (less than 30 s for each trajectory path analyzed in this study), making it possible to cluster thousands of genes from many cells without the need to limit clustering to only a few hundred of genes as in [4]. Although scSTEM still needs some “prefiltering” process to reduce the list of input genes to a few thousand (3000 ~ 5000 in this study), the clustering engine can easily handle larger number of genes. However, to generate interpretable results, we recommend the users to limit to a few thousand of input genes. Following clustering, the user can also compare significant profiles across clusters to identify pathways and processes that differ between different paths. scSTEM performs comparison by testing the number of overlapping genes in clusters from different paths. Users can then compare the expression patterns of clusters with significant overlap and combine with other evidence (GO enrichment, cell type information, etc.) to determine whether there are functional relevancies between clusters. An important assumption used by scSTEM is that the expression change of a gene along the edges between milestones is linear. There is some prior work that supports this idea for time series expression data [21]. We have also further examined whether such linearity is observed for genes in the human fetal immune cell data set. Genes expressed in at least 5% of the cells that mapped to the edges were selected for this analysis. For each gene, we used corrected *p*-values (BH corrections) from F test to evaluate the fit of a linear model. As shown in Additional File 2: Fig. S11, we observe a significant fit for a linear model for over 50% of the genes for internal (non-terminal) edges. We observe less agreement for terminal edges, but this is in part a function of the way the trajectory is constructed. Terminal edges have much fewer cells and significant than internal edges (since each branching further splits the number of genes) which leads to noisy expression profile that is harder to model using a simple curve. As a method for sanity check, we have added the relevant functionality in scSTEM interface, where users can conveniently generate plot that shows linear fit for a trajectory path.

While useful, there are several limitations that should be noted. First, scSTEM relies on trajectory inference methods, which may not accurately reconstruct cell trajectories. While the package provides access to several such methods, and so it is likely that at least a few will work well, there is no guarantee that the resulting trajectories indeed capture the underlying process. Second, most trajectory inference methods do not reflect directionality of progression along the path. scSTEM uses real time point information to pinpoint the origin of the trajectory tree, but if such time information does not agree with the real direction, the analysis would be inaccurate. Third, in some cases, trajectory methods provide too many branches, which artificially increases the path length and may be detrimental to scSTEM analysis.

To further improve scSTEM in the future, time point information may be explicitly used during the construction of trajectory and clustering of genes. Methods that provide directionality of single-cell progression such as RNA velocity [22, 23] may be considered.

We have provided scSTEM as an R shiny GUI-based tool which does not require any coding experience (available at https://github.com/alexQiSong/scSTEM). While not being limited to bioinformatians and computational biologists, we aim to provide scSTEM to the broader audiences of all researchers interested in analyzing scRNA-seq data.

## Conclusions

We have developed a computational tool, scSTEM, to cluster genes for scRNA-seq data. We applied scSTEM to published scRNA-seq data sets, and the results showed that scSTEM has successfully captured the T cell, NK cell, immune process, and neuron development-related gene clusters. The comparison between gene clusters from T cell and NK cell trajectory path has revealed NK cell-specific expression patterns of several genes. scSTEM is able to work with different trajectory inference methods for single-cell data. Users may choose the method of interest and identify the best method for the relevant biological question. We believe scSTEM can be used as a powerful tool to dissect complex scRNA-seq datasets and reveal meaningful gene expression patterns.

## Methods

### Retrieval and preprocessing of data sets

We have tested scSTEM on three time series single-cell data sets: (1) human fetal immune cells [13], (2) mouse embryonic blood cells [11], (3) mouse embryonic neural crest cells [11]. For the human fetal immune cell data set, we downloaded processed UMI counts from https://descartes.brotmanbaty.org/bbi/human-gene-expression-during-development/. The data set contained 103,766 cells sampled at 18 time points. For this data set, we used the top 3000 cell-type-specific marker genes as was done in the original publication [13]. For mouse embryonic blood cells and mouse embryonic neural crest cells, we downloaded processed UMI counts from https://oncoscape.v3.sttrcancer.org/atlas.gs.washington.edu.mouse.rna/downloads. The mouse embryonic blood cell dataset contained 42,262 cells sampled at 5 time points while the neural crest dataset contained 22,283 cells and 5 time points. For both datasets, we filtered genes expressed in less than 10 cells and cells expressing less than 200 genes. We next identified the top 5000 most variable genes by Seurat (Version 4.0.3) and used these genes for downstream analysis.

### scSTEM pipeline

#### Normalization and dimensionality reduction

For initial visualization of the data, scSTEM performs normalization of UMI count data using log normalization provided by Monocle3 package [11]. Normalized data is reduced to 100 dimensions prior to dimensionality reduction by UMAP.

#### Selection of cells by partition

Instead of using all cells to perform trajectory inference, it is important to infer trajectory tree for a subset of cells relevant to the biological question of interest. Therefore, scSTEM allows users to select a subset of cells after the dimensionality reduction step and before trajectory inference step. scSTEM uses Leiden algorithm to cluster cells. For each connected component in the Leiden graph, the software assigns a cluster ID allowing the users to analyze each of these separately. User can then select the cell partition by selecting the corresponding cluster IDs in the scSTEM GUI and perform trajectory inference for the selected cells.

#### Trajectory inference

To perform pseudotime inference, scSTEM allows users to select one of several popular methods. To enable this, we use a general trajectory inference framework provided by the dynverse package [2]. Dynverse supports several popular methods including slingshot [7], PAGA [12], and more. The trajectory is represented by a graph in which each node is a milestone node and edges connecting them represent transitions between two milestone nodes. Although scSTEM is primarily designed to work with general trajectory structures, it imposes some constraints which impacts the set of methods that can be used for the initial pseudotime inference. These include (1) the inferred trajectories provided by the method should follow a tree structure without loops; (2) the inference method only takes a set of starting cells as prior information if prior information is required. These requirements still leave a very large set of 19 trajectory inference methods which can be used with scSTEM (See Additional file 2: Table S1).

Trajectory inference will produce a trajectory tree, with nodes representing possible cell states and edges representing the transitions among these cell states. Following the terminologies used by Dynverse, we term these nodes as “milestone nodes.” A path is the shortest path connecting the root node and a leaf node. The goal for scSTEM is to perform gene clustering on each path. scSTEM assigns an ID number to each path. The user can then select a single or multiple path(s) by checking the corresponding path ID numbers in the GUI.

#### Summarizing gene expression levels along a path

While each path may contain hundreds or even thousands of cells, the changes between milestone nodes is usually linear for genes. Thus, the trajectory of a specific gene along a specific path can be represented with a small set of values, one for each of the milestone nodes along the paths. These values are used as inputs to scSTEM to cluster the genes along the path and to assign significance value to the clusters. However, there are several different ways in which one can summarize the expression of genes along the path and different summarization may be more appropriate for different studies.

scSTEM provides three methods for aggregating expression levels from cells assigned to a specific segment. A segment is defined as the set of milestone nodes and edges connecting two consecutive milestone nodes in the trajectory. We define three types of milestone nodes: (1) the root node, (2) branching node, and (3) leaf node. For directed trees, a branching node is a node having greater than or equal to two outgoing edges. For undirected tree, branching node is a node having greater than or equal to three edges connected to it. Once trajectory tree has been constructed, scSTEM will iterate over all possible paths going from root node to leaf node and aggregate gene expressions in each key segment and in each path. We term these segments as “sampled time point,” and each path may include several such sampled time points. scSTEM then clusters genes for each path based on these time point values. When the number of sampled time points is less than three, the STEM algorithm will not be able to produce meaningful clusters. To address this issue, in such cases scSTEM further breaks paths into more segments using ordering of pseudotime values. For paths containing only one key segment, scSTEM will partition the segment into three segments by looking for 1/3 and 2/3 quantile points. For paths containing only two key segments, scSTEM will partition each segment into two segments by looking for median point. We have observed that this strategy is useful for trajectory inference methods that do not create complex branch structure. The metrics used to aggregate gene expressions for each sampled time point are described below.

##### Mean expression

This metric simply computes the mean of normalized expression values for each gene in all cells belonging to a sampled time point.

##### Entropy reduction score

ROGUE (Ratio of Global Unshifted Entropy) is a statistic initially proposed to measure the impurity of cell population [24]. We used ROGUE score to measure the purity of a gene in a cell population. The ROGUE score for gene *i* in a population of *n* cells is calculated as

$$d{s}_{i}=\mathit{ln}E\left({X}_{i}\right)-\frac{{\sum }_{j=1}^{n}\left(\mathit{ln}{X}_{ij}\right)}{n}$$

where $E\left({X}_{i}\right)$ is the expected gene count from Poisson-Gamma model and $\mathit{ln}E\left({X}_{i}\right)$ represents differential entropy under ${H}_{0}$ that cell population is homogeneous and thus only one Poisson-Gamma component is present for all cells. The right part of the equation $\frac{{\sum }_{j=1}^{n}\left(\mathit{ln}{X}_{ij}\right)}{n}$ measures the differential entropy for the other extreme case: when cell population is extremely heterogeneous, each cell represents its own cluster. This metric can capture the consistency of expression levels for this gene in this segment.

##### Change rate of gene expression

This metric measures the rate of expression change for each gene along the segment. To compute the change rate, we first apply a filtering strategy to remove zero and low expression values which may represent dropouts. We use a *Z*-score based simple strategy here. For each gene, scSTEM computes *Z*-scores for all expressions in the segment and then removes cells with *Z*-score < 1.96 and *Z*-score > 1.96 for that time point. After this filtering step, scSTEM fits a linear model using the remaining cells:

$${e}_{i}={\upbeta }_{\mathrm{i}}t+\varepsilon$$

where ${{\varvec{e}}}_{{\varvec{i}}}$ denotes expression of gene *i*, *t* is the pseudotime values of cells within each sampled time point, and *ε* represents the noise in the expression values. scSTEM then uses ${\beta }_{i}$, the linear rate parameter, as the value for this gene in this segment.'

### STEM clustering

STEM is an algorithm for clustering genes in short time series data [10]. STEM clusters genes by assigning genes to pre-generated model profiles that represent distinct expression profiles. The main advantage of STEM over other unsupervised methods is the use of a pre-defined set of profiles which enables it to assign significance to each of the clusters. Each gene is assigned to one of these pre-defined profiles based on its expression level, and clusters are constructed from genes assigned to the same profile (or a number of different profiles with a similar trajectory). Since the set of possible profiles is pre-determined, STEM can assign significance to the number of genes assigned to each profile by using permutation tests. We used metrics described in “Methods” to summarize the expression of each gene in each of the paths. Values are ordered for each gene based on the pseudotime order of the edges on a path leading to a short time series dataset that summarizes the expression of the genes in each selected path. STEM further processes the input to compute log ratio for each sampled time point with respect to the first sampled time point and then uses these ratios to cluster genes. See also [9, 10] for more details about STEM algorithm.

### Comparison of clustering results in different paths

STEM can compare the clustering results from two different bulk RNA-seq time series data sets. Similarly, we have enabled scSTEM to perform comparison of clustering results from different trajectory paths to reveal how functional gene clusters change along different cell lineages. scSTEM performs hypergeometric test to identify clusters that have significant number of overlapping genes in different paths then visualizes the expression patterns of these similar clusters (Fig. 1B). Users can easily perform the comparison in the scSTEM GUI by selecting any two paths of interest.

### GO enrichment analysis

We used built-in GO enrichment analysis in STEM software to perform GO analysis. If input gene IDs are ensemble IDs, scSTEM will first map them to gene symbols by biomaRt R package then passed down the converted IDs to STEM for GO analysis.

### Comparison of different clustering and DE methods

For DE gene analysis, we first performed trajectory inference with Monocle 3 and extracted cells mapped to path 1 (Fig. 2B) from the human fetal immune cell data set. Then, we ran differential expression analysis using graph_test() function in Monocle 3 and the workflow of DE gene analysis in tradeSeq (see tradeSeq documentation: http://www.bioconductor.org/packages/release/bioc/vignettes/tradeSeq/inst/doc/Monocle.html). GO enrichment was then performed using ranking of *p*-values.

For clustering analysis, we have run two comparison methods as well, scHot and tradeSeq, on the same path. scHOT clusters genes by computing the pairwise Spearman correlation followed by hierarchical clustering. Such operations are very computationally expensive for large datasets. We thus only applied scHOT to the top 300 marker genes in the NK cell path (the path shown in Fig. 2B).

## Supplementary Information

Additional file 1. Gene cluster sizes, profile sizes, and p-values for each combination of trajectory inference method and gene summarization metric; Number of NK cell markers for the clusters presented in Fig. 5.

Additional file 2. All supplementary notes, supplementary figures, and supplementary tables.

Additional file 3. Gene IDs for clusters presented in Fig. 5.

Additional file 4. GO enrichment analysis results for clusters presented in Fig. 5.

Additional file 5. Review history.

## Data Availability

The datasets generated and/or analyzed during the current study are available in the GitHub repository: https://github.com/alexQiSong/scSTEM_sample_data/ [25]. The original scRNA-seq data (processed as gene count matrices) for human fetal immune cells can be accessed at descartes.brotmanbaty.org [13], and the corresponding raw data can be accessed through dbGaP under the accession number phs002003.v1.p1. The original scRNA-seq data (processed as gene count matrices) for mouse embryonic blood cells and mouse embryonic neural crest cells can be accessed at https://oncoscape.v3.sttrcancer.org/atlas.gs.washington.edu.mouse.rna/landing [11], and the corresponding raw data can be accessed through NCBI Gene Expression Omnibus under the accession number GSE119945. scSTEM package, the analysis code, and other processed data used in this study are available at GitHub repository: https://github.com/alexQiSong/scSTEM under MIT license [25], and at Zenodo. (https://doi.org/10.5281/zenodo.6331254) [26] under Creative Commons Attribution 4.0 International license.
