# Supplementary material for: scSTEM: clustering pseudotime ordered single-cell data
Source: Genome Biol. 2022 Jul 7;23:150. doi: 10.1186/s13059-022-02716-9 (PMC9264648; doi:10.1186/s13059-022-02716-9)
Supplement: Supplementary file 2 — Additional file 2. All supplementary notes, supplementary figures, and supplementary tables. [file 13059_2022_2716_MOESM2_ESM.pdf]

Additional file 2 – Supplementary Information

scSTEM: Clustering pseudo-time ordered single cell data

Qi Song[1], Jingtao Wang[2], Ziv Bar-Joseph[13*]

[1]Computational Biology Department, School of Computer Science, Carnegie Mellon University, Pittsburgh, PA 15213, USA

[2]Department of Medicine, Division of Experimental Medicine, McGill University, Montreal, Quebec, Canada

[3]Machine Learning Department, School of Computer Science, Carnegie Mellon University, Pittsburgh, PA 15213, USA

* To whom correspondence should be addressed.

Corresponding author: Ziv Bar-Joseph: zivbj@cs.cmu.edu

Supplementary Note

Generation of synthetic scRNA-seq datasets.

Differentially expressed genes (DEGs) are widely used to characterize the transcriptomic signatures of genes in different cellular states and are considered as driver genes that lead to different cell fates. An interesting question to ask is whether the significant clusters detected by scSTEM can capture these biological signals. While the gold-standard datasets of DEGs are lacking, we generated synthetic scRNA-seq datasets with simulated DEGs and predefined trajectory topologies. These datasets were generated using dyntoy tool included in the dynverse toolkit (https://github.com/dynverse/dyntoy). Dyntoy can generate scRNA-seq data with prespecified trajectory topology and number of DEGs. Briefly, dyntoy starts simulation by generating a predefined trajectory topology structure and sampling cells along the edges between milestone nodes. Then for each gene, density of each cell is estimated by bivariate normal distribution, with mean and standard deviation sampled from 2D cell positions. Next, if a gene is selected to be a DEG, count value for that gene will be generated using zero-inflated negative binomial distribution with distribution mean scaled by the estimated cell density values. Otherwise count value will be scaled by randomly shuffled cell density values. Based on the normalized count matrix, we used "calculate_pseudotime()" function in dynverse toolkit to obtain pseudotime values for all cells. We followed this procedure and generated synthetic scRNA-seq datasets for three types of trajectory: linear, bifurcating, and multifurcating. For each type, we generated expression data matrix of 10000 cells and 3000 genes with varying percentages of DEGs (10%, 30%, 50%). This yielded 9 synthetic datasets in total.

Evaluation of scSTEM using synthetic scRNA-seq data

We compared genes of the significant clusters identified by scSTEM to the simulated DEGs. The results, as shown in Additional file 2: Fig S12, indicate that all genes in scSTEM-identfied clusters are from the simulated DEGs. Though several simulated DE genes were not part of the significant clusters, this is a function of the way the DE genes are generated and not related to the ability of scSTEM to infer significant trajectories. To show that this is indeed the case we further partitioned the DEGs into two sets. We further partitioned the DEGs into two sets: 1) common genes, defined as those DE genes assigned to significant scSTEM clusters and 2) DEG-specifc genes, which are DEGs not identified by scSTEM. We computed absolute values of Pearson correlation coefficients between the expressions of each gene set and their corresponding cell pseudotime. Results, presented in Additional file2: Fig. S12 show that while common genes exhibit high correlation with cell pseudotime, DE genes not assigned to scSTEM clusters are much less correlated with the cell pseudotime. Thus, scSTEM can correctly identify all clusters composed of temporally changing genes, indicating that DEGs identified by scSTEM include stronger biological signals than other DEGs.

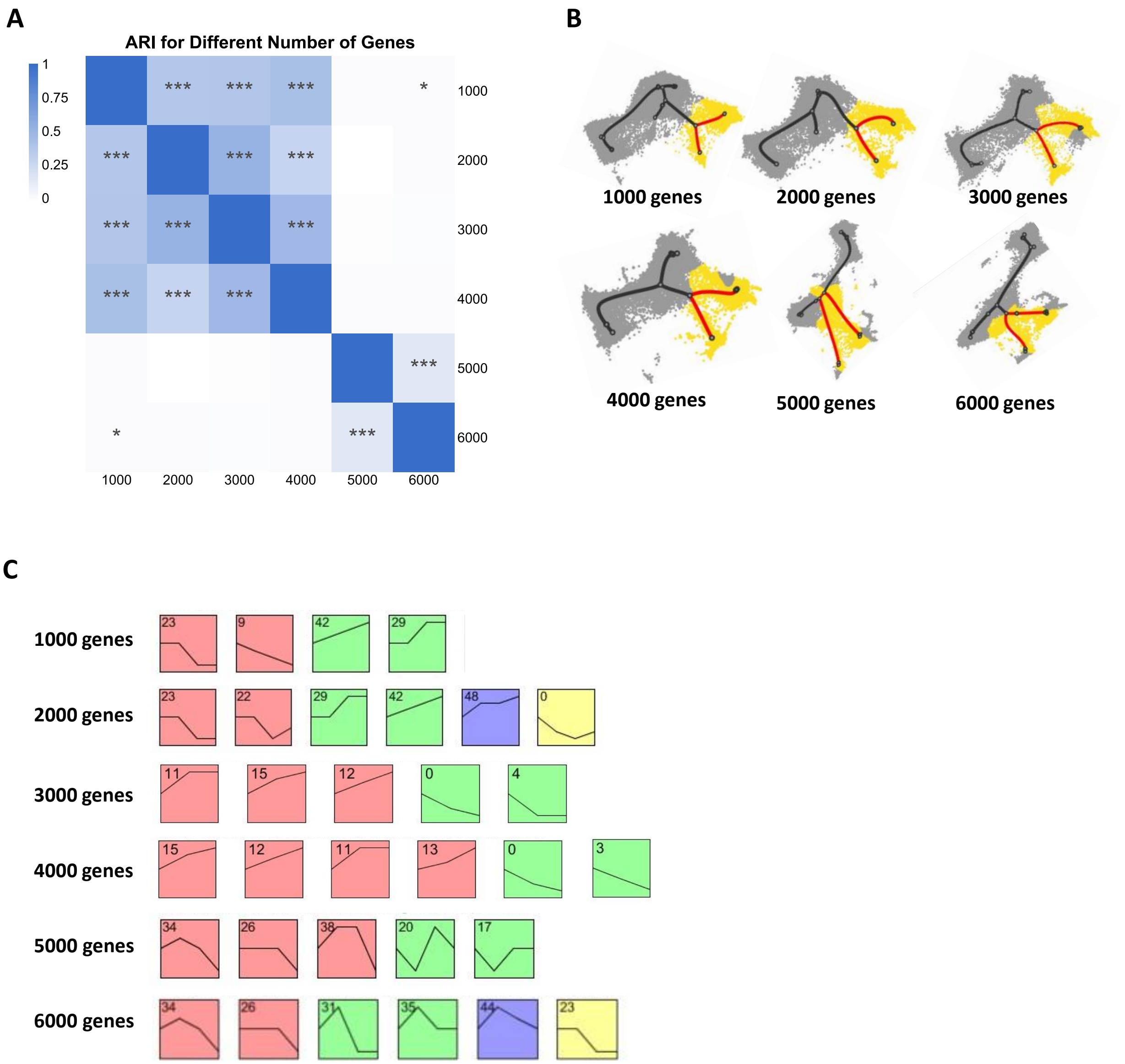

Figure S1. Clustering results for various input gene sizes. A The heatmap showing the adjusted rand index (ARI) for each comparison of two different input sizes. Rows and columns were marked by corresponding number of input genes. Significance for each cell was computed based on an empirical distribution of ARI score. P-values were adjusted by Benjamini-Hochberg correction. '***': p-value < 0.001; '**': 0.001 < p-value < 0.01 and '*': 0.01 < p-value < 0.05. **B** Trajectory of NK cell related path for different input settings. **C** Cluster visualizations for different input settings.

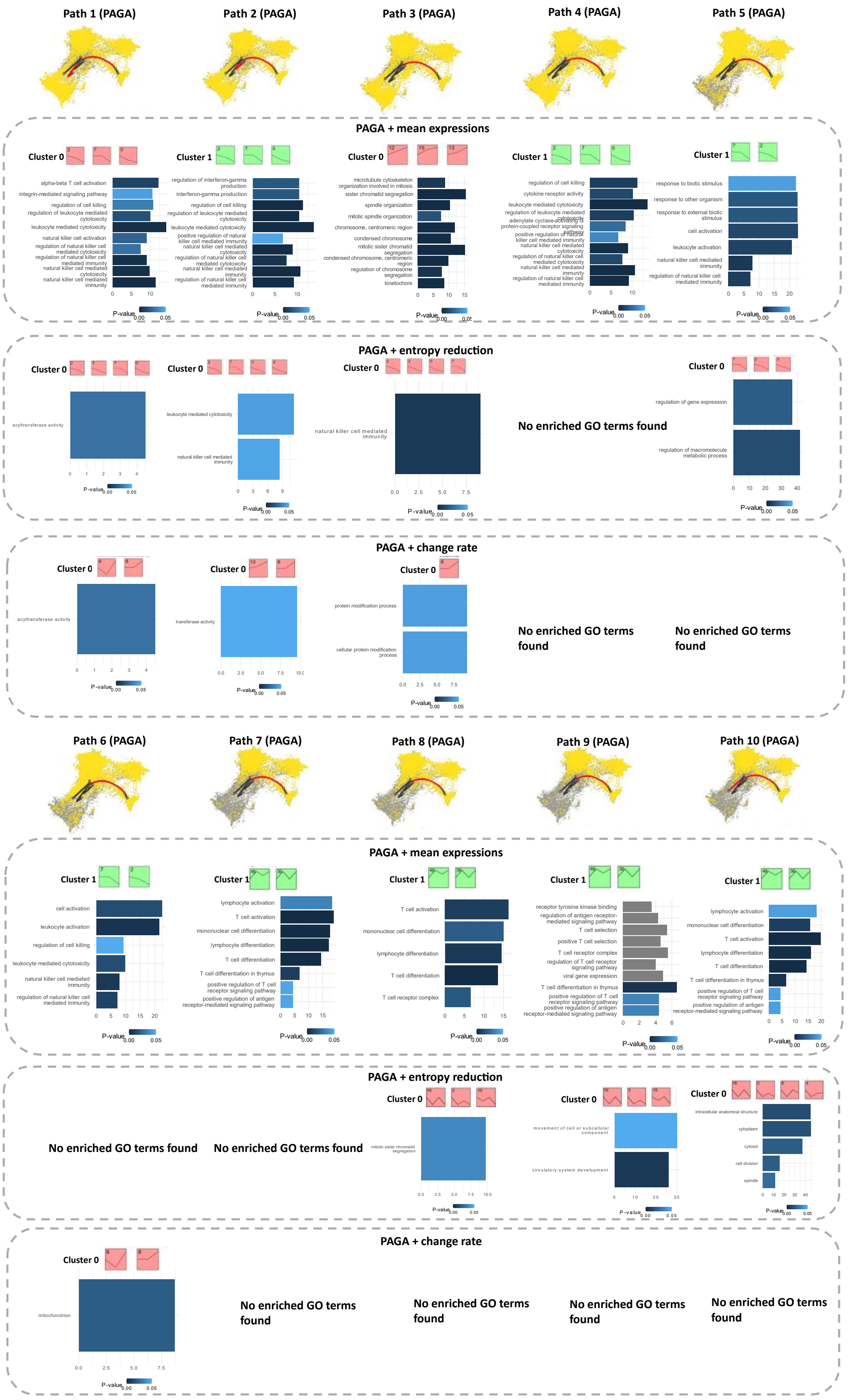

Path 1 (PAGA)
Path 2 (PAGA)
Path 3 (PAGA)
Path 4 (PAGA)
Path 5 (PAGA)
PAGA + mean expressions
Cluster 0
Cluster 1
Cluster 0
Cluster 1
Cluster 1
alpha-beta T cell activation
integrin-mediated signaling pathway
regulation of cell killing
regulation of leukocyte mediated cytotoxicity
leukocyte mediated cytotoxicity
natural killer cell activation
regulation of natural killer cell mediated cytotoxicity
regulation of natural killer cell mediated immunity
natural killer cell mediated cytotoxicity
natural killer cell mediated immunity
regulation of interferon-gamma production
interferon-gamma production
regulation of cell killing
regulation of leukocyte mediated cytotoxicity
leukocyte mediated cytotoxicity
positive regulation of natural killer cell mediated immunity
regulation of natural killer cell mediated cytotoxicity
natural killer cell mediated immunity
regulation of natural killer cell mediated immunity
microtubule cytoskeleton organization involved in mitosis
sister chromatid segregation
spindle organization
mitotic spindle organization
chromosome, centromeric region
condensed chromosome
mitotic sister chromatid segregation
condensed chromosome, centromeric region
regulation of chromosome segregation
kinetochore
regulation of cell killing
cytokine receptor activity
leukocyte mediated cytotoxicity
regulation of leukocyte mediated cytotoxicity
natural killer cell mediated cytotoxicity
regulation of natural killer cell mediated cytotoxicity
natural killer cell mediated immunity
regulation of natural killer cell mediated immunity
response to biotic stimulus
response to other organism
response to external biotic stimulus
cell activation
leukocyte activation
natural killer cell mediated immunity
regulation of natural killer cell mediated immunity
P-value
PAGA + entropy reduction
Cluster 0
acyltransferase activity
leukocyte mediated cytotoxicity
natural killer cell mediated immunity
No enriched GO terms found
regulation of gene expression
regulation of macromolecule metabolic process
PAGA + change rate
acyltransferase activity
transferase activity
protein modification process
cellular protein modification process
No enriched GO terms found
No enriched GO terms found
Path 6 (PAGA)
Path 7 (PAGA)
Path 8 (PAGA)
Path 9 (PAGA)
Path 10 (PAGA)
PAGA + mean expressions
cell activation
leukocyte activation
regulation of cell killing
leukocyte mediated cytotoxicity
natural killer cell mediated immunity
regulation of natural killer cell mediated immunity
lymphocyte activation
T cell activation
mononuclear cell differentiation
lymphocyte differentiation
T cell differentiation
T cell differentiation in thymus
positive regulation of T cell receptor signaling pathway
positive regulation of antigen receptor-mediated signaling pathway
T cell receptor complex
receptor tyrosine kinase binding
regulation of antigen receptor-mediated signaling pathway
T cell selection
positive T cell selection
regulation of T cell receptor signaling pathway
viral gene expression
PAGA + entropy reduction
No enriched GO terms found
No enriched GO terms found
mitotic sister chromatid segregation
movement of cell or subcellular component
circulatory system development
intracellular anatomical structure
cytoplasm
cytosol
cell division
spindle
PAGA + change rate
mitochondrion
No enriched GO terms found
No enriched GO terms found
No enriched GO terms found
No enriched GO terms found

Figure S2. Results from PAGA + all gene summarization methods for human fetal immune cell data set. Black curves indicate the trajectory tree, and the highlighted red curves are edges along one selected path. Yellow cells are cells mapped to the selected path and grey cells are other remaining cells. The barplots represent top 10 enriched GO terms in the corresponding clusters, ranked by enrichment fold. Y axis in barplots indicate the corresponding GO term name and X axis in barplots indicate the counts of genes annotated with the corresponding term in the cluster. Above the barplots are the visualizations of expression pattern for the corresponding clusters.

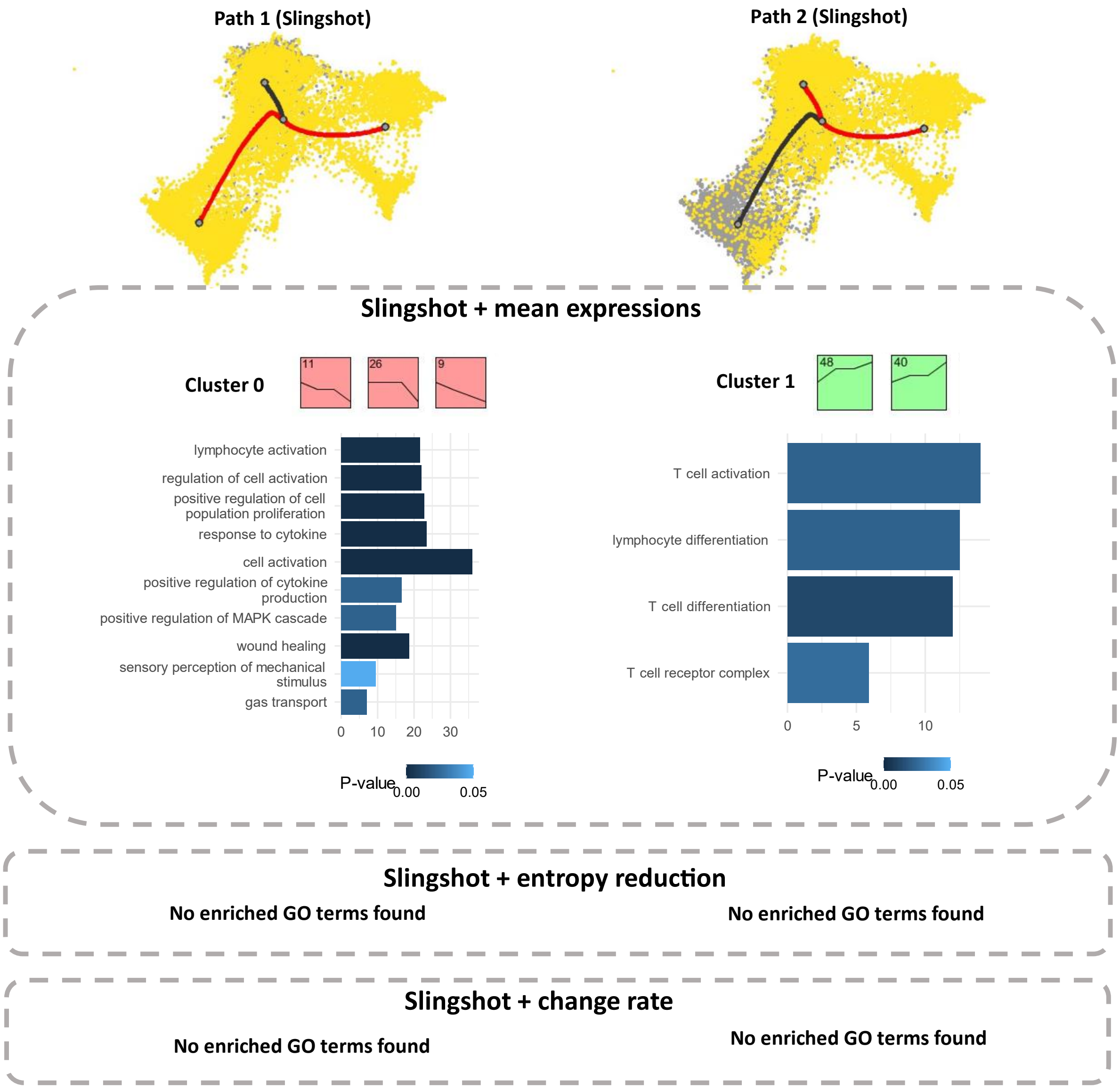

Figure S3. Results from Slingshot + all gene summarization methods for human fetal immune cell data set. Black curves indicate the trajectory tree, and the highlighted red curves are edges along one selected path. Yellow cells are cells mapped to the selected path and grey cells are other remaining cells. The barplots represent top 10 enriched GO terms in the corresponding clusters, ranked by enrichment fold. Y axis in barplots indicate the corresponding GO term name and X axis in barplots indicate the counts of genes annotated with the corresponding term in the cluster. Above the barplots are the visualizations of expression pattern for the corresponding clusters.

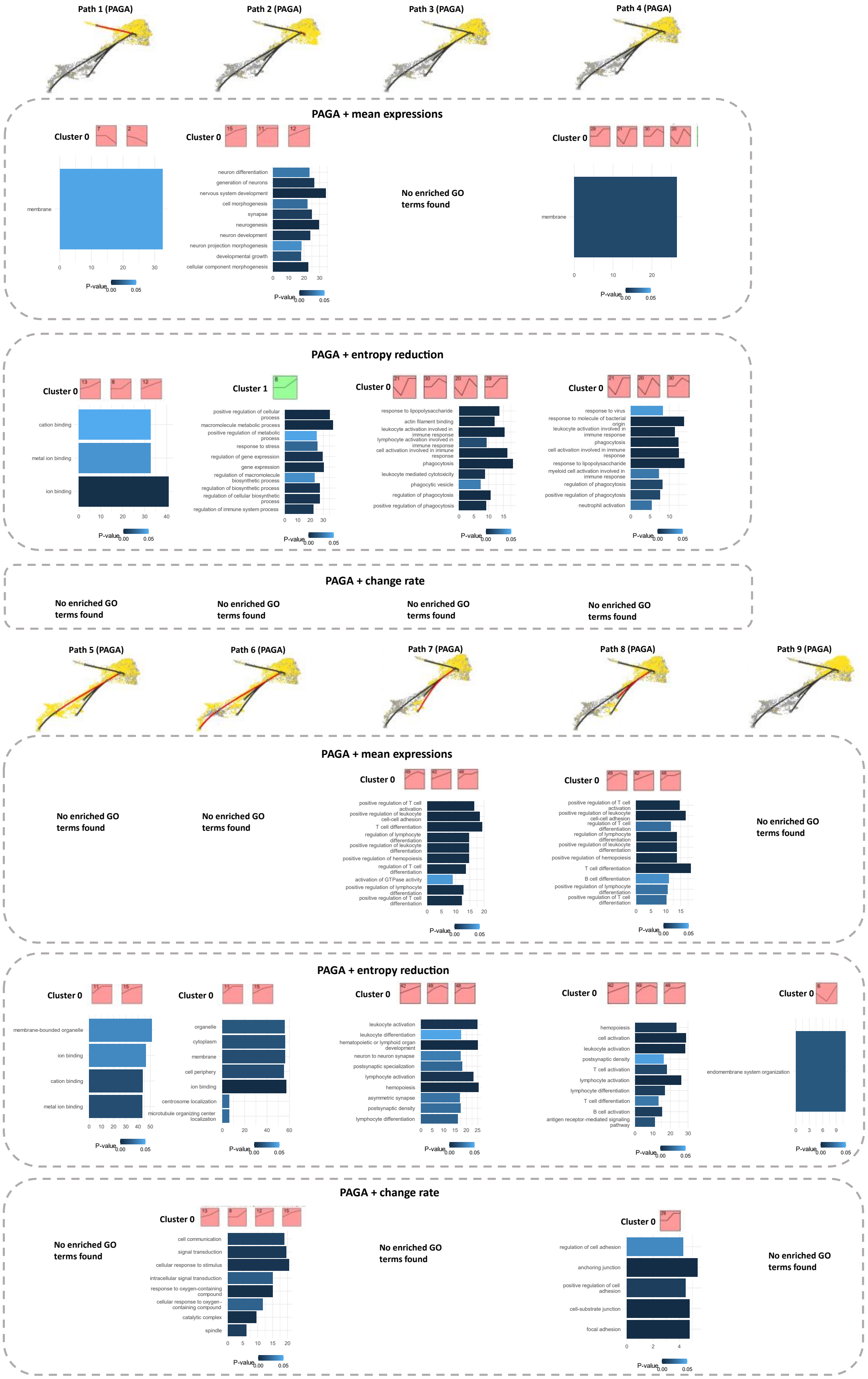

Path 1 (PAGA)
Path 2 (PAGA)
Path 3 (PAGA)
Path 4 (PAGA)
PAGA + mean expressions
Cluster 0
Cluster 0
Cluster 0
membrane
neuron differentiation
generation of neurons
nervous system development
cell morphogenesis
synapse
neurogenesis
neuron development
neuron projection morphogenesis
developmental growth
cellular component morphogenesis
No enriched GO terms found
membrane
P-value
PAGA + entropy reduction
Cluster 0
Cluster 1
Cluster 0
Cluster 0
cation binding
metal ion binding
ion binding
positive regulation of cellular process
macromolecule metabolic process
positive regulation of metabolic process
response to stress
regulation of gene expression
gene expression
regulation of macromolecule biosynthetic process
regulation of biosynthetic process
regulation of cellular biosynthetic process
regulation of immune system process
response to lipopolysaccharide
actin filament binding
leukocyte activation involved in immune response
lymphocyte activation involved in immune response
cell activation involved in immune response
phagocytosis
leukocyte mediated cytotoxicity
phagocytic vesicle
regulation of phagocytosis
positive regulation of phagocytosis
response to virus
response to molecule of bacterial origin
leukocyte activation involved in immune response
phagocytosis
cell activation involved in immune response
response to lipopolysaccharide
myeloid cell activation involved in immune response
regulation of phagocytosis
positive regulation of phagocytosis
neutrophil activation
PAGA + change rate
No enriched GO terms found
No enriched GO terms found
No enriched GO terms found
No enriched GO terms found
Path 5 (PAGA)
Path 6 (PAGA)
Path 7 (PAGA)
Path 8 (PAGA)
Path 9 (PAGA)
PAGA + mean expressions
No enriched GO terms found
No enriched GO terms found
Cluster 0
Cluster 0
No enriched GO terms found
positive regulation of T cell activation
positive regulation of leukocyte cell-cell adhesion
T cell differentiation
regulation of lymphocyte differentiation
positive regulation of leukocyte differentiation
positive regulation of hemopoiesis
regulation of T cell differentiation
activation of GTPase activity
positive regulation of lymphocyte differentiation
positive regulation of T cell differentiation
positive regulation of T cell activation
positive regulation of leukocyte cell-cell adhesion
regulation of T cell differentiation
regulation of lymphocyte differentiation
positive regulation of leukocyte differentiation
positive regulation of hemopoiesis
T cell differentiation
B cell differentiation
positive regulation of lymphocyte differentiation
positive regulation of T cell differentiation
PAGA + entropy reduction
Cluster 0
Cluster 0
Cluster 0
Cluster 0
Cluster 0
membrane-bounded organelle
ion binding
cation binding
metal ion binding
organelle
cytoplasm
membrane
cell periphery
ion binding
centrosome localization
microtubule organizing center localization
leukocyte activation
leukocyte differentiation
hematopoietic or lymphoid organ development
neuron to neuron synapse
postsynaptic specialization
lymphocyte activation
hemopoiesis
asymmetric synapse
postsynaptic density
lymphocyte differentiation
hemopoiesis
cell activation
leukocyte activation
postsynaptic density
T cell activation
lymphocyte activation
lymphocyte differentiation
T cell differentiation
B cell activation
antigen receptor-mediated signaling pathway
endomembrane system organization
PAGA + change rate
No enriched GO terms found
Cluster 0
cell communication
signal transduction
cellular response to stimulus
intracellular signal transduction
response to oxygen-containing compound
cellular response to oxygen-containing compound
catalytic complex
spindle
No enriched GO terms found
Cluster 0
regulation of cell adhesion
anchoring junction
positive regulation of cell adhesion
cell-substrate junction
focal adhesion
No enriched GO terms found

Figure S4. Results from PAGA + all gene summarization methods for mouse embryonic blood cell data set. Black curves indicate the trajectory tree, and the highlighted red curves are edges along one selected path. Yellow cells are cells mapped to the selected path and grey cells are other remaining cells. The barplots represent top 10 enriched GO terms in the corresponding clusters, ranked by enrichment fold. Y axis in barplots indicate the corresponding GO term name and X axis in barplots indicate the counts of genes annotated with the corresponding term in the cluster. Above the barplots are the visualizations of expression pattern for the corresponding clusters.

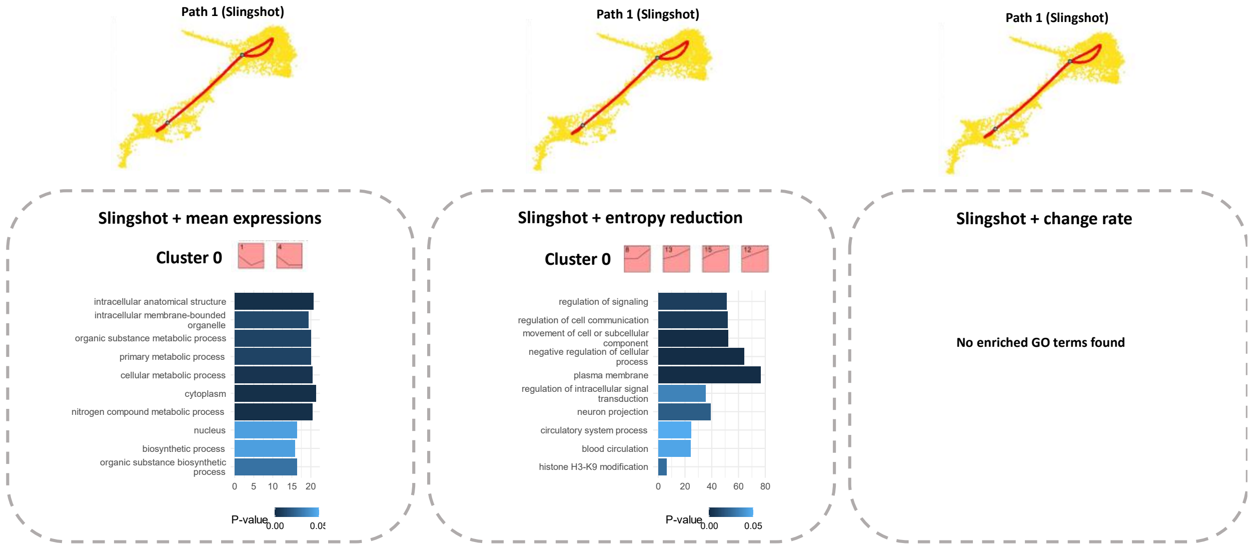

Figure S5. Results from Slingshot + all gene summarization methods for mouse embryonic blood cell data set. Black curves indicate the trajectory tree, and the highlighted red curves are edges along one selected path. Yellow cells are cells mapped to the selected path and grey cells are other remaining cells. The barplots represent top 10 enriched GO terms in the corresponding clusters, ranked by enrichment fold. Y axis in barplots indicate the corresponding GO term name and X axis in barplots indicate the counts of genes annotated with the corresponding term in the cluster. Above the barplots are the visualizations of expression pattern for the corresponding clusters.

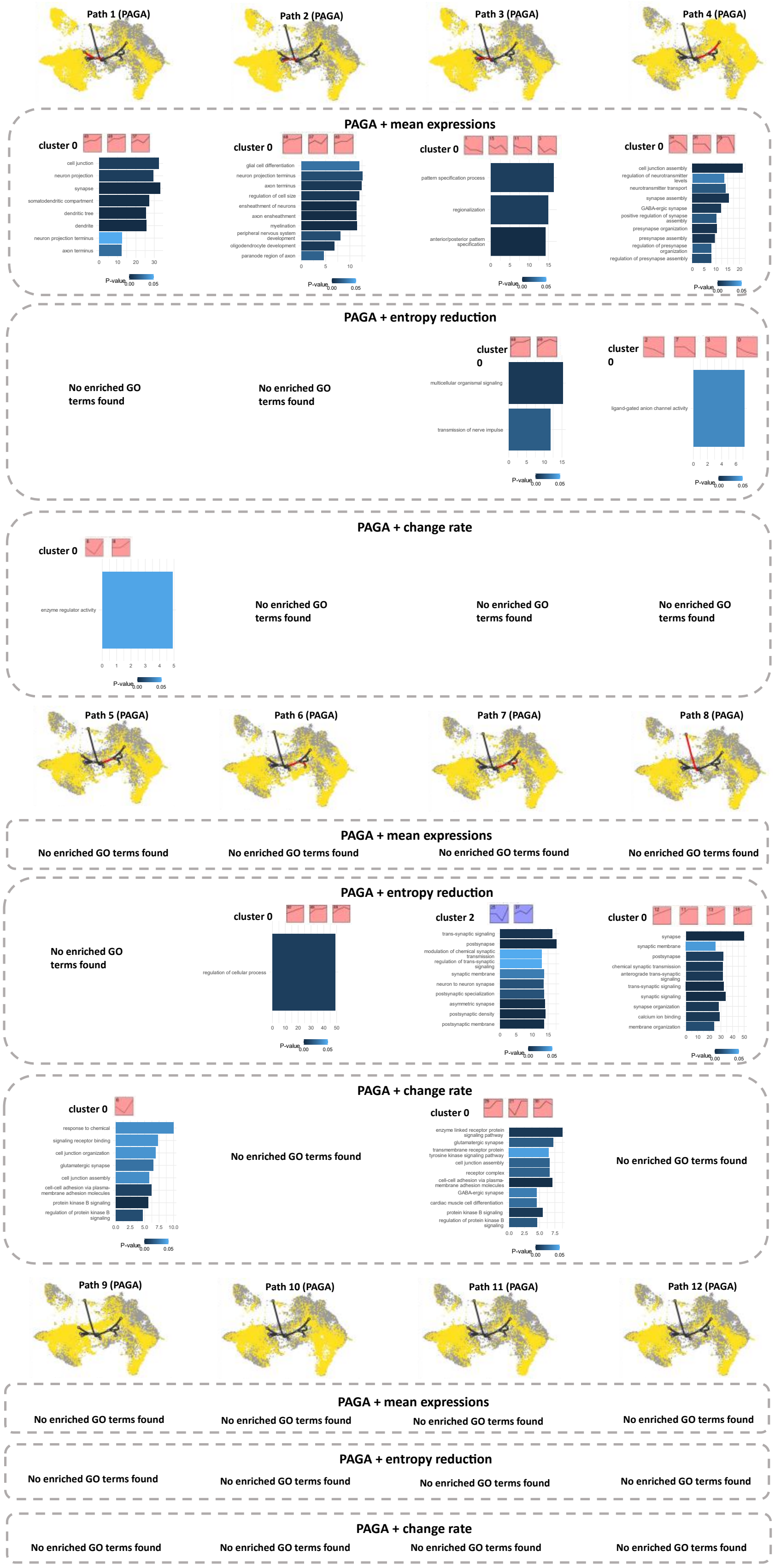

Path 1 (PAGA)
Path 2 (PAGA)
Path 3 (PAGA)
Path 4 (PAGA)
PAGA + mean expressions
cluster 0
cluster 0
cluster 0
cluster 0
PAGA + entropy reduction
No enriched GO terms found
No enriched GO terms found
cluster 0
cluster 0
PAGA + change rate
cluster 0
No enriched GO terms found
No enriched GO terms found
No enriched GO terms found
Path 5 (PAGA)
Path 6 (PAGA)
Path 7 (PAGA)
Path 8 (PAGA)
PAGA + mean expressions
No enriched GO terms found
No enriched GO terms found
No enriched GO terms found
No enriched GO terms found
PAGA + entropy reduction
No enriched GO terms found
cluster 0
cluster 2
cluster 0
PAGA + change rate
cluster 0
No enriched GO terms found
cluster 0
No enriched GO terms found
Path 9 (PAGA)
Path 10 (PAGA)
Path 11 (PAGA)
Path 12 (PAGA)
PAGA + mean expressions
No enriched GO terms found
No enriched GO terms found
No enriched GO terms found
No enriched GO terms found
PAGA + entropy reduction
No enriched GO terms found
No enriched GO terms found
No enriched GO terms found
No enriched GO terms found
PAGA + change rate
No enriched GO terms found
No enriched GO terms found
No enriched GO terms found
No enriched GO terms found

Figure S6. Results from PAGA + all gene summarization methods for mouse neural crest data set. Black curves indicate the trajectory tree, and the highlighted red curves are edges along one selected path. Yellow cells are cells mapped to the selected path and grey cells are other remaining cells. The barplots represent top 10 enriched GO terms in the corresponding clusters, ranked by enrichment fold. Y axis in barplots indicate the corresponding GO term name and X axis in barplots indicate the counts of genes annotated with the corresponding term in the cluster. Above the barplots are the visualizations of expression pattern for the corresponding clusters.

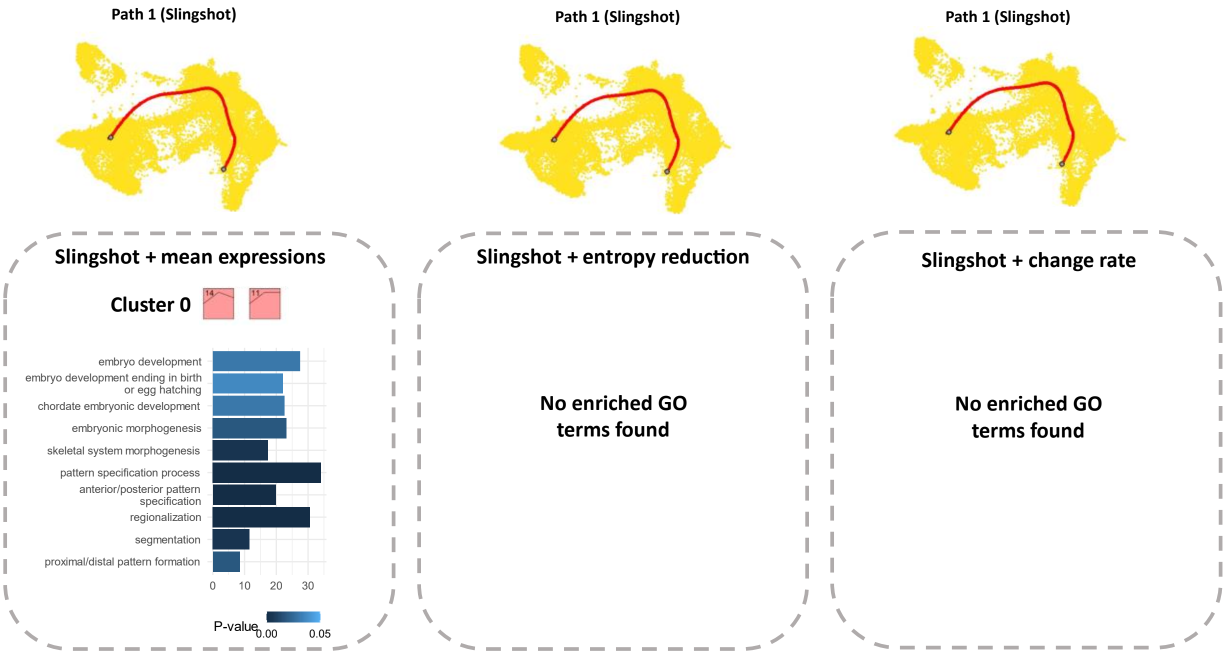

Figure S7. Results from Slingshot + all gene summarization methods for mouse neural crest data set. Black curves indicate the trajectory tree, and the highlighted red curves are edges along one selected path. Yellow cells are cells mapped to the selected path and grey cells are other remaining cells. The barplots represent top 10 enriched GO terms in the corresponding clusters, ranked by enrichment fold. Y axis in barplots indicate the corresponding GO term name and X axis in barplots indicate the counts of genes annotated with the corresponding term in the cluster. Above the barplots are the visualizations of expression pattern for the corresponding clusters.

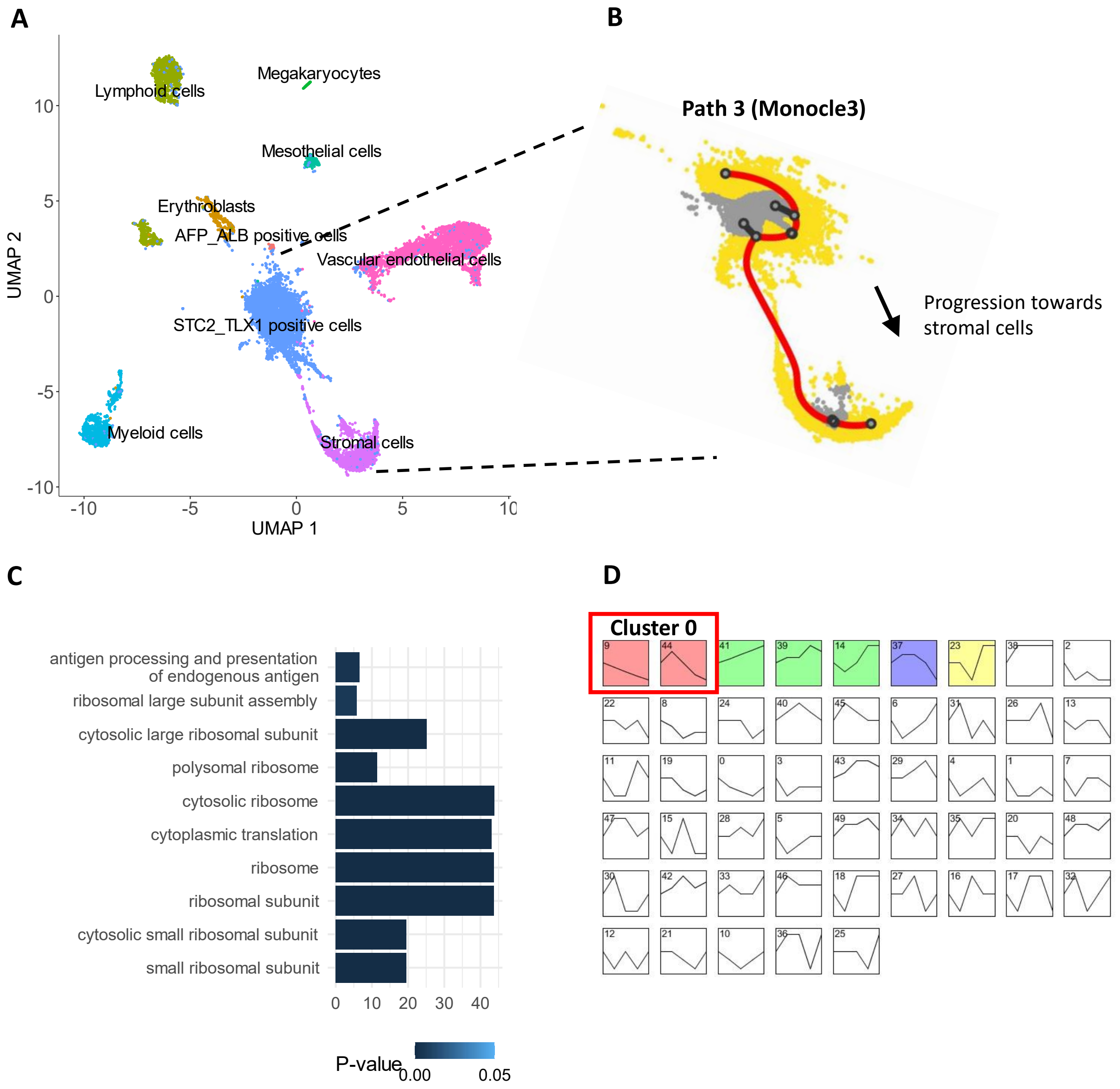

Figure S8. scSTEM results for STC2_TLX1 and stromal cell cluster. A UMAP visualization of all spleen cells. **B** Trajectory of path 3 inferred by Monocle3. Black curves indicate the trajectory tree, and the highlighted red curves are edges along path 3. Yellow cells are cells mapped to path 3 and grey cells are other remaining cells. **C** Top enriched GO terms in cluster 3, ranked by enrichment fold. Antigen presentation related term was ranked at the top. Y axis marks the corresponding GO term name and X axis marks the counts of genes annotated with the corresponding term in the cluster. **D** Visualization of expression profiles for each cluster. Highlighted in red box are the gene profiles in cluster 0.

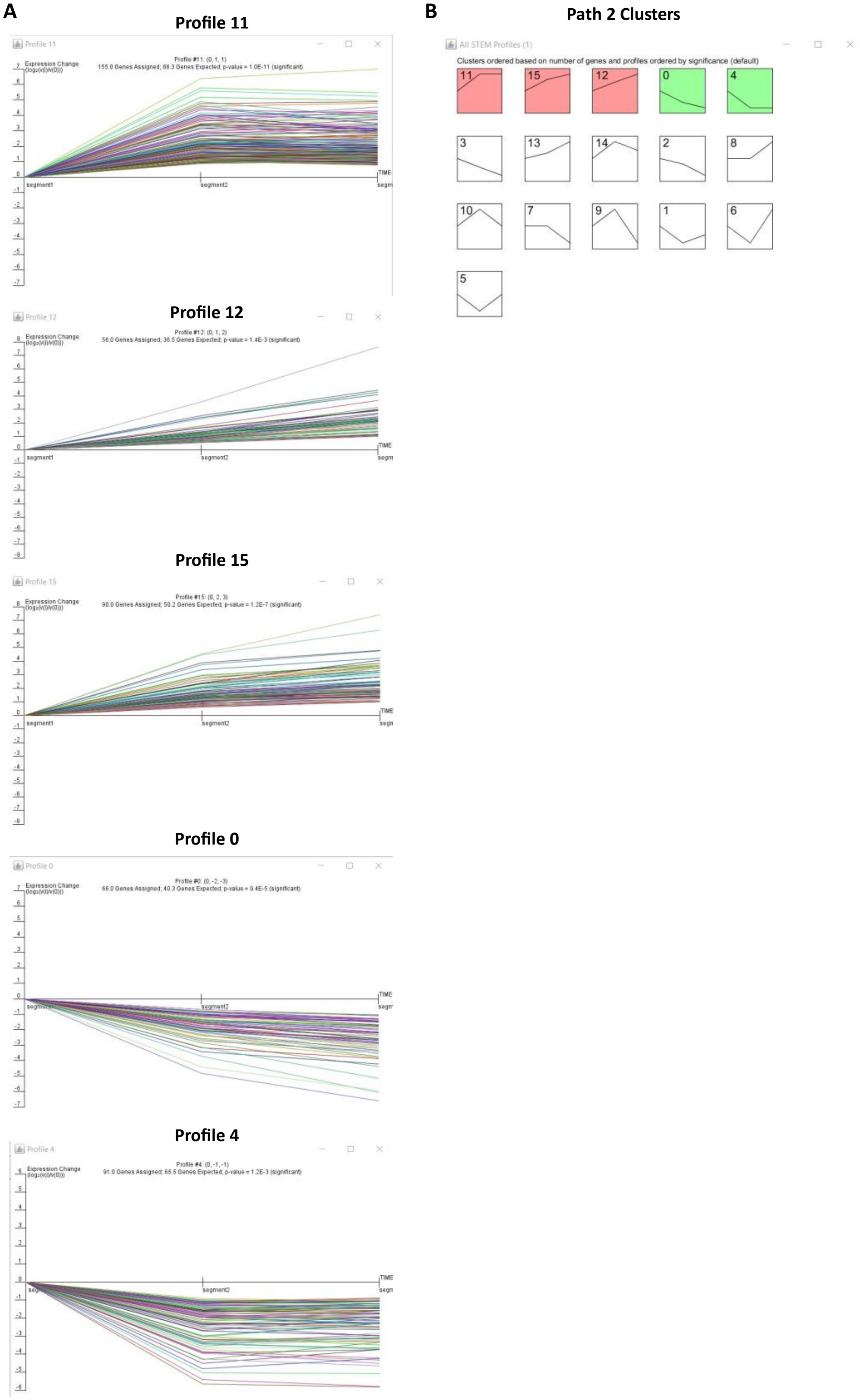
A
Profile 11
Profile #11: (0, 1, 1)
155.0 Genes Assigned; 88.3 Genes Expected; p-value = 1.0E-11 (significant)
Expression Change
segment1
segment2
TIME
Profile 12
Profile #12: (0, 1, 2)
56.0 Genes Assigned; 36.5 Genes Expected; p-value = 1.4E-3 (significant)
Profile 15
Profile #15: (0, 2, 3)
90.0 Genes Assigned; 50.2 Genes Expected; p-value = 1.2E-7 (significant)
Profile 0
Profile #0: (0, -2, -3)
66.0 Genes Assigned; 40.3 Genes Expected; p-value = 9.4E-5 (significant)
Profile 4
Profile #4: (0, -1, -1)
91.0 Genes Assigned; 65.5 Genes Expected; p-value = 1.2E-3 (significant)
B
Path 2 Clusters
All STEM Profiles (1)
Clusters ordered based on number of genes and profiles ordered by significance (default)
11
15
12
0
4
3
13
14
2
8
10
7
9
1
6
5

Figure S9. Detailed gene profiles for the significant clusters in path 2 from human fetal immune cell data set. A the detailed expression pattern of each gene fitted to the profiles presented in B**.** Profile number at the top of each plot in A corresponds to the profile numbers shown in B**. B** All clusters and gene profiles identified in path 2. Gene profiles assigned to the same cluster were marked by the same color.

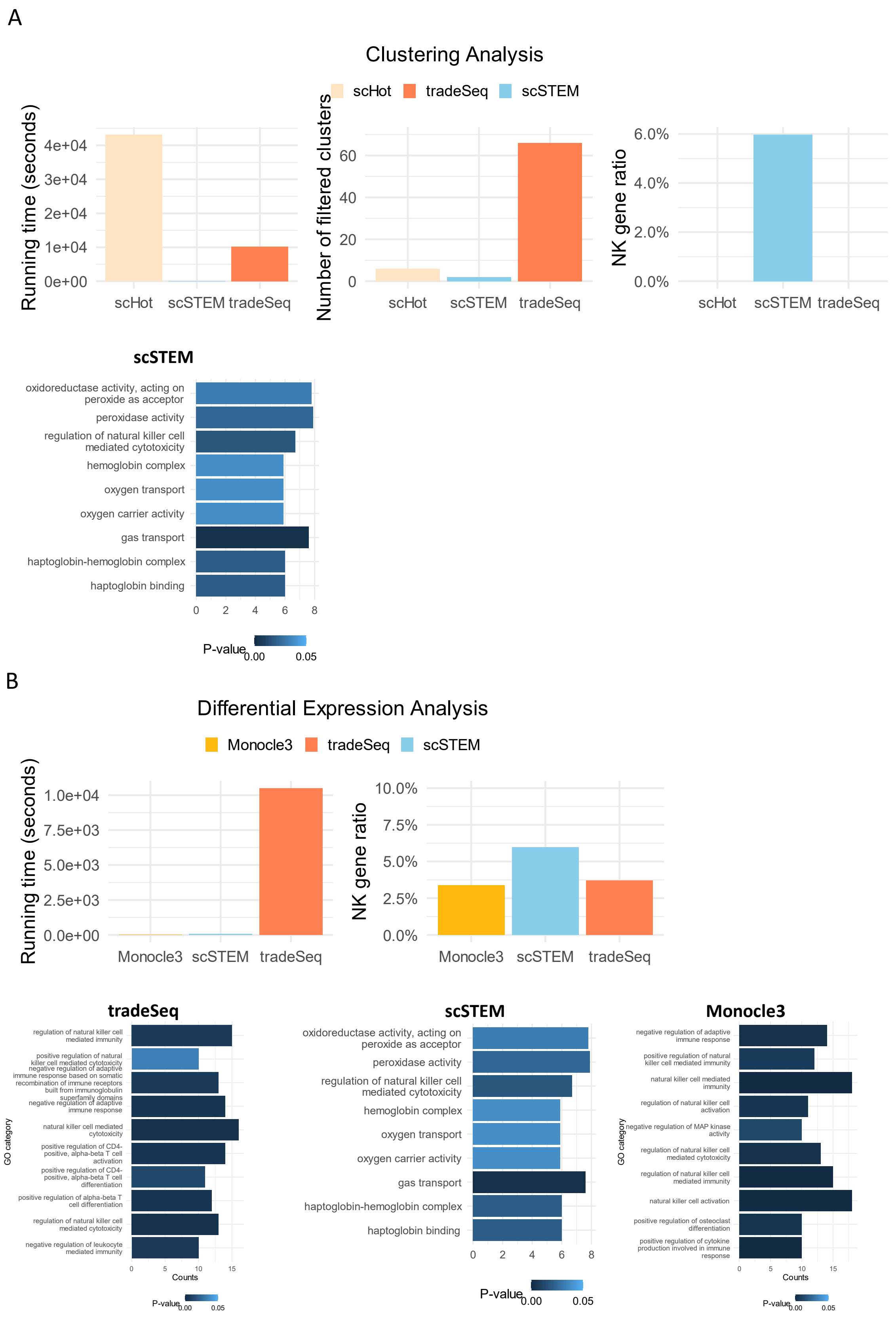
A
Clustering Analysis
scHot
tradeSeq
scSTEM
Running time (seconds)
4e+04
3e+04
2e+04
1e+04
0e+00
scHot
scSTEM
tradeSeq
Number of filtered clusters
60
40
20
0
NK gene ratio
6.0%
4.0%
2.0%
0.0%
scSTEM
oxidoreductase activity, acting on peroxide as acceptor
peroxidase activity
regulation of natural killer cell mediated cytotoxicity
hemoglobin complex
oxygen transport
oxygen carrier activity
gas transport
haptoglobin-hemoglobin complex
haptoglobin binding
0
2
4
6
8
P-value
0.00
0.05
B
Differential Expression Analysis
Monocle3
tradeSeq
scSTEM
Running time (seconds)
1.0e+04
7.5e+03
5.0e+03
2.5e+03
0.0e+00
NK gene ratio
10.0%
7.5%
5.0%
2.5%
0.0%
tradeSeq
regulation of natural killer cell mediated immunity
positive regulation of natural killer cell mediated cytotoxicity
negative regulation of adaptive immune response based on somatic recombination of immune receptors built from immunoglobulin superfamily domains
negative regulation of adaptive immune response
natural killer cell mediated cytotoxicity
positive regulation of CD4-positive, alpha-beta T cell activation
positive regulation of CD4-positive, alpha-beta T cell differentiation
positive regulation of alpha-beta T cell differentiation
regulation of natural killer cell mediated cytotoxicity
negative regulation of leukocyte mediated immunity
GO category
0
5
10
15
Counts
P-value
0.00
0.05
scSTEM
oxidoreductase activity, acting on peroxide as acceptor
peroxidase activity
regulation of natural killer cell mediated cytotoxicity
hemoglobin complex
oxygen transport
oxygen carrier activity
gas transport
haptoglobin-hemoglobin complex
haptoglobin binding
0
2
4
6
8
P-value
0.00
0.05
Monocle3
negative regulation of adaptive immune response
positive regulation of natural killer cell mediated immunity
natural killer cell mediated immunity
regulation of natural killer cell activation
negative regulation of MAP kinase activity
regulation of natural killer cell mediated cytotoxicity
regulation of natural killer cell mediated immunity
natural killer cell activation
positive regulation of osteoclast differentiation
positive regulation of cytokine production involved in immune response
GO category
0
5
10
15
Counts
P-value
0.00
0.05

Figure S10. Benchmark and evaluation for different clustering methods and DE methods. A top: comparison between scSTEM and other clustering methods. Comparison metrics are marked along the Y axis in each plot. Bottpm: the top 10 enriched GO terms in NK related clusters from scSTEM. Other methods failed to identify any NK related GO terms enriched in the top five largest clusters and thus GO results are not shown. **B** Comparison between scSTEM and other DE methods. Comparison metrics are marked along the Y axis in each plot. Bottom: the top 10 enriched GO terms in NK related clusters for scSTEM and the top 10 enriched GO terms for DE genes from tradeSeq and Monocle3.

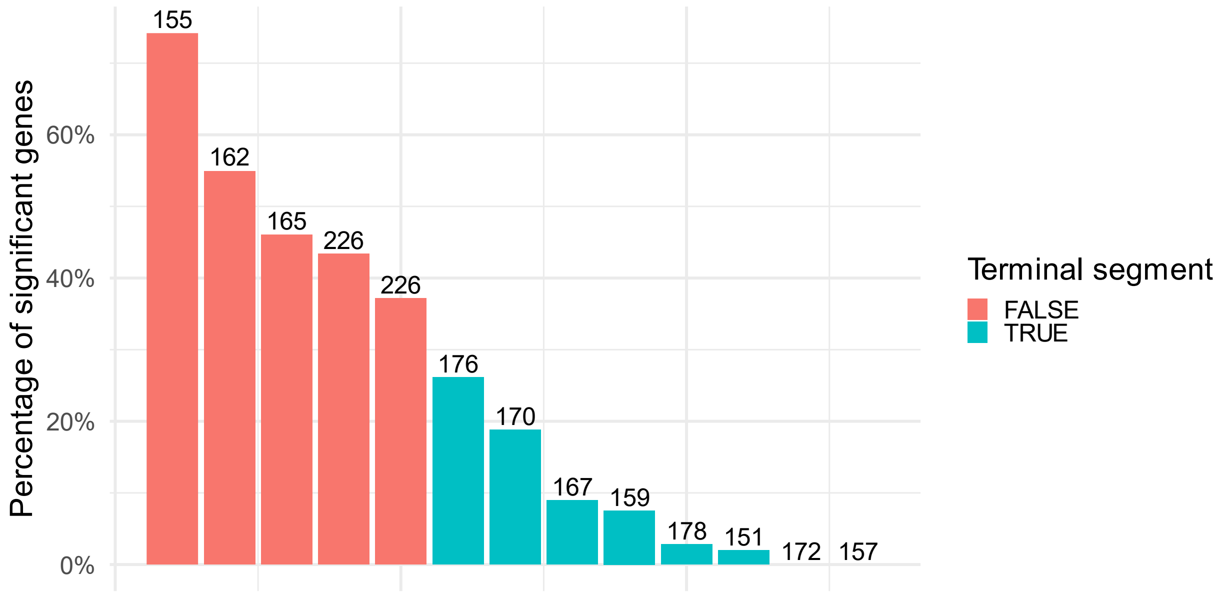

Figure S11. Percentage of significant genes from F-test for linear models. Each bar represents a segment of trajectory path. A segment represents a set of edges between two root/branching milestone nodes. Colors indicate whether the corresponding segment is at the terminal of the

corresponding trajectory path. Total number of genes after filtering is marked at the top of each bar.

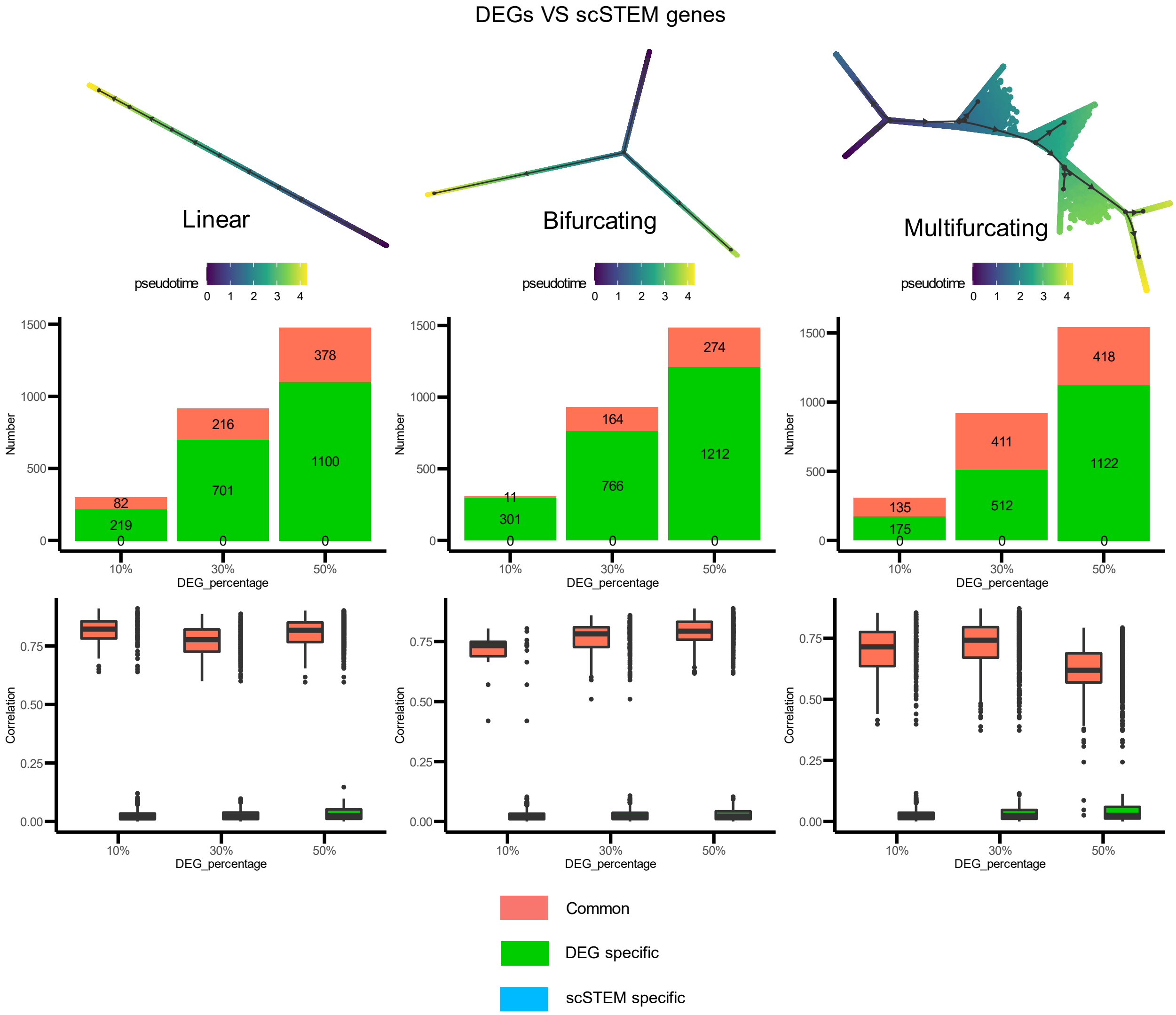

Figure S12. scSTEM VS DEGs on simulated scRNA-seq datasets. Top panel: example of the trajectory type in each simulation.; **Middle panel**: Number of genes in each category. "common" represents common genes between scSTEM-identified significant cluster genes and simulated DEGs; "scSTEM-specific" represents genes identified by scSTEM but not found in simulated DEGs; "DEG-specific" represents simulated DEGs not identified by scSTEM. For each simulation, five datasets were generated using different percentage of DEGs, which is indicated in the horizontal axis. **Bottom panel**: correlations between each category of genes (as represented in the middle panel) and cell pseudotime.

Table S1. Inference methods supported by scSTEM.

Method Name	Method Name	Method Name
Slingshot	PAGA Tree	PAGA
SLICE	pCreode	CellTree maptpx
SCUBA	CellTree vem	Sincell
RaceID / StemID	ElPiGraph	CellTrails
URD	CellRouter	CellTree Gibbs
SLICER	CALISTA	Monocle 2 (Monocle DDRTree)
Monocle 3		
