# Supplementary material for: scSTEM: clustering pseudotime ordered single-cell data
Source: Genome Biol. 2022 Jul 7;23:150. doi: 10.1186/s13059-022-02716-9 (PMC9264648; doi:10.1186/s13059-022-02716-9)
Supplement: Supplementary file 5 — Additional file 5. Review history. [file 13059_2022_2716_MOESM5_ESM.docx]

Review History

**First round of review**

**Reviewer 1**

**Were you able to assess all statistics in the manuscript, including the appropriateness of statistical tests used?**

Yes, and I have assessed the statistics in my report.

**Were you able to directly test the methods?**

No.

**Comments to author:**

Here, Song et al present scSTEM which is a software for clustering pseudo-time ordered single cell data. Both clustering and pseudo time ordering are popular approaches for analyzing single-cell data. However, they can often be considered complementary and thus this approach for analyzing single cell data by clustering genes is potentially very valuable to the community. The ability to identify useful trajectories could be very valuable to many researchers analyzing complex datasets. Consequently, scSTEM could potentially be of value to the community. Overall, the manuscript is well written and the structure is easy to follow.

I have the following major concerns:

1. The GitHub page for the project looks very nice and it appears as if the documentation is at the level that one would expect today. However, despite the apparent high quality, I had major issues installing the software on a Linux system. In the end, I got stuck at

> renv::restore(project = install_folder, lockfile = paste0(install_folder,"renv.lock"), prompt = F)

* The library is already synchronized with the lockfile.

> renv::activate(install_folder)

* Project '~/scstem' loaded. [renv 0.14.0]

Warning message:

In renv_load_r(project, lockfile$R) :

missing required [R] section in lockfile

> library("scSTEM")

Error in library("scSTEM") : there is no package called 'scSTEM'

> run_scstem_GUI()

Error in run_scstem_GUI() : could not find function "run_scstem_GUI"

I also asked another (more savvy) member of my group who tried to install on three different platforms (to Linux and one Windows) without any success. Thus, an improved troubleshooting section is needed as well as more informative error messages.

2. Fig 2 and S1-2 highlight the identification of immune related GO processes. However, I would like to know how many other processes were identified? In this case, T cell development serves as a useful positive control, but if scSTEM also reports a large number of other GO processes then it will be much more challenging to use in a setting where we do not know as well what to expect. The same type of question applies to the other case studies in the manuscript.

3. The authors have not mentioned the scHOT method (https://www.nature.com/articles/s41592-020-0885-x) which appears to be able to also identify clusters of genes from a trajectory and highlights enriched GO terms. Could you please comment on this method and if relevant also benchmark the two.

4. I am probably missing something obvious here, but I do not understand the difference between the top and bottom plots in the right hand side of Fig 5a. Why is cluster 45 highlighted at the top, but not at the bottom? It is my understanding that the genes it contains are the same, no? Or does the small addition at the end of path 7 make a big difference?

5. Also, for this example you have only highlighted the differences that serve as positive controls. I would like to know what other groups of genes that scSTEM picks out for this example as that will give a better idea of what it will be like to use the tool in a situation where less is known about the outcome.

In the example for fig 5, the scenario that you consider is essentially: "Find gene clusters and check which ones are overrepresented for NK cells". What I would like to see is a use case which is somewhat more realistic. In many cases, one would need to compare to lots of marker gene lists or GO terms to figure out what is going on. I would like the authors to present a use case where the interpretation and downstream analysis is more challenging and extensive than what is currently the case.

6. When two paths are compared as in Fig 5, do you use any multiple hypothesis correction for the tests?

7. How easy is it for a future user to modify scSTEM to allow it to use another trajectory inference method?

Minor issue:

1 On line 346 there is a reference to 2.2.3 but it is not clear what this corresponds to.

2 I do not really understand where path 2 with 300 genes comes from in fig 5. Looking at line 4 in the additional_file1 I see a path2 with 301 genes - is this a typo or a misunderstanding from my side? Same with path6 where I find one with 56 genes, but for path7 it seems to be correct with 124 genes.

**Reviewer 2**

**Were you able to assess all statistics in the manuscript, including the appropriateness of statistical tests used?**

Yes, and I have assessed the statistics in my report.

**Were you able to directly test the methods?**

Yes.

**Comments to author:**

Summary

In this manuscript, the authors describe a method called scSTEM. This is an adaptation of their previously published STEM method to scRNA seq data. The paper gives a clear overview of the scSTEM algorithm, both the original STEM method and the extensions required for scRNA-Seq. The authors use 3 real datasets to showcase usages of scSTEM. They are quite convincing in demonstrating the usefulness of the method: by providing p-values to cluster assignments, the method simplifies biological interpretation and allows for comparisons between branches. They also provide software on github that is very easy to use and flexible to upstream analysis choices. The shiny app in particular provides a very nice UI that will be useful for non-experienced coders.

scSTEM will be very useful to the community, provided some additions to the paper. In particular, the paper makes several assumptions when extending STEM to scRNA-Seq data which are not properly demonstrated, and it lacks comparisons with appropriate methods to demonstrate superiority.

Major comments

- The authors assert (Line 291-292) that "the changes between milestone nodes is usually linear for genes". However, they do not back this claim by either appropriate reference, or demonstration on real datasets.

- Each milestone node is mapped to a time point (figure 1 for example) and then each gene is associated with a profile. However, this induces a transformation between pseudotime and the time of the profiles, so that

the distance (in pseudotime) between each milestone node becomes the same. While this won't matter at the level of a single path, it might be inappropriate to do so when comparing various paths. Milestone nodes

might have very different pseudotimes after branching: this time wrapping may artificially create or remove differences between paths, and the authors need to demonstrate that it is not the case.

- No comparisons to other methods are made, especially for 2 things.

+ the common workflow to identify GO terms enriched in a branch (or between branches) is to fit a trajectory, then find DE genes using methods such as those implemented in monocle 2 or 3, GPfates, or tradeSeq

and then use the ranking of p-values as input to GO methods.

+ ImpulseDE (ImpulseDE2) similarly fits profiles to genes (with an impulse model). The authors need to explain how scSTEM differs from this.

- While the paper is very strong in terms of real data, it is lacking simulations. The converse, already used by the authors, provides a suitable simulation framework.

Minor comments

- Filtering, as reported by the authors, can have a large impact on clustering results, although scSTEM might be less impacted. Some sensitivity analyses and comparison with usual clustering methods would help show

this.

- Related, in Line 183, the authors perform a hypergeometric test using all genes as background. This is incorrect since none-filtered genes cannot be in the clusters. The background should be all genes that pass

filtering.

- It would be interesting to show how some genes fit to their assigned profile, for a few clusters, as a supplementary figure.

- Some case studies have too many lineages, especially with monocle 3 which tend to find lots of small lineages that are data artifacts. Tuning the parameters will remove those and produce a more likely trajectory.

- Can the comparison between branches be extended to more than two paths?

- Are the authors planning to submit the package to CRAN or Bioconductor? This would increase its diffusion (with the understanding that package with shiny apps are hard to register)

- The author did provide the scSTEM package and the data but failed to provide the code needed to reproduce the paper.

**Authors Response**

**Point-by-point responses to the reviewers’ comments:**

Reviewer #1:

Here, Song et al present scSTEM which is a software for clustering pseudo-time ordered single cell data. Both clustering and pseudo time ordering are popular approaches for analyzing single-cell data. However, they can often be considered complementary and thus this approach for analyzing single cell data by clustering genes is potentially very valuable to the community. The ability to identify useful trajectories could be very valuable to many researchers analyzing complex datasets. Consequently, scSTEM could potentially be of value to the community. Overall, the manuscript is well written and the structure is easy to follow.

I have the following major concerns:

1. The GitHub page for the project looks very nice and it appears as if the documentation is at the level that one would expect today. However, despite the apparent high quality, I had major issues installing the software on a Linux system. In the end, I got stuck at

> renv::restore(project = install_folder, lockfile = paste0(install_folder,"renv.lock"), prompt = F)

* The library is already synchronized with the lockfile.

> renv::activate(install_folder)

* Project '~/scstem' loaded. [renv 0.14.0]

Warning message:

In renv_load_r(project, lockfile$R) :

missing required [R] section in lockfile

> library("scSTEM")

Error in library("scSTEM") : there is no package called 'scSTEM'

> run_scstem_GUI()

Error in run_scstem_GUI() : could not find function "run_scstem_GUI"

I also asked another (more savvy) member of my group who tried to install on three different platforms (to Linux and one Windows) without any success. Thus, an improved troubleshooting section is needed as well as more informative error messages.

*Response: We apologize for the inconvenience during the installation. We tested the method prior to submission and have also asked several members of our group to download and test it and it worked fine on several different machines and operating systems. Still, we further looked into the error the reviewer mentioned and were indeed able to successfully reproduce it. The problem was caused by the renv.lock file that does not include certain fields for specifying some basic packages which may have not been previously installed by the reviewer. We have now updated the renv.lock file and installation instructions accordingly. Please try to install the latest version in GitHub repository.*

2. Fig 2 and S1-2 highlight the identification of immune related GO processes. However, I would like to know how many other processes were identified? In this case, T cell development serves as a useful positive control, but if scSTEM also reports a large number of other GO processes then it will be much more challenging to use in a setting where we do not know as well what to expect. The same type of question applies to the other case studies in the manuscript.

*Response: Thank you for providing constructive feedback regarding GO results. To address this comment we have now updated our main figures and supplementary figures to list the top 10 significantly enriched GO terms for each cluster. As can be seen in the revised main figures, we were able to again identify strong implications of immune process related GO terms from human immune cell data set and mouse blood cell data set. For example, in Fig 2B GO terms for the NK cell path include “regulation of natural killer cell meidated cytotoxity” and in Fig 2C and 2D GO terms for T cell path include “T cell activation” and “T cell differentiation”. Similarly, for mouse blood cell data set the GO terms include “phagocytic vesicle” and“phatocytosis” (Fig 3B and Fig 3C) and leukocyte/lymphocyte related GO terms (Fig 3C and 3D). This likely indicates that the functional role of white blood cells was strengthening during cell state transitions. Additonally, as seen in Fig 2, scSTEM has only identified a few significant GO terms and these correctly capture the most relevant categoires (T cell and NK cell related terms). This makes it easy to intepret the cluster functions. For clusters with more GO terms identfied, our ranking strategy can also extract useful functional information from GO analysis results.*

3. The authors have not mentioned the scHOT method (https://www.nature.com/articles/s41592-020-0885-x) which appears to be able to also identify clusters of genes from a trajectory and highlights enriched GO terms. Could you please comment on this method and if relevant also benchmark the two.

*Response: Thank you for mentioning the scHOT method. Following this comment we have tested scHOT on the data we presented in the paper. We first note that scHOT dos not scale to the number of cells and genes in the dataset that scSTEM is applied to. Specifically, scHOT clusters genes by computing pairwise spearman correlation followed by hierarchical clustering. Such operations are very computationally expensive for large dataset and would take days for the entire datasets we analyzed. We thus only applied scHOT to the top 300 marker genes in the NK cell path (as in Fig. 2A). We compared the results to scSTEM using GO functional enrichment as the criteria See Fig. S1 for the evaluation results. As can be seen, unlike scSTEM, scHOT was not able to identify NK cell related GO terms as enriched in any of the gene clusters it identified.*

4. I am probably missing something obvious here, but I do not understand the difference between the top and bottom plots in the right hand side of Fig 5a. Why is cluster 45 highlighted at the top, but not at the bottom? It is my understanding that the genes it contains are the same, no? Or does the small addition at the end of path 7 make a big difference?

*Response: While both top and bottom clusters are labeled as ‘45’ and have the same profile (since this is STEM numbering and the numbering of the profiles is the same for all paths) they are actually from *different* paths. Specifically, the top figure is from path 7 and the bottom from path 6. The genes assigned to each of them may also be different since they are from only partially overlapping cells. In this case, path 6 and 7 overlap in the first 3 time points, but not in the fourth, and so the expression of genes assigned in each case may be different. Specifically, there are 57 genes in profile 45 for path 6 (C2P6) and 56 genes in profile 45 in path 7 (C4P7) at the bottom. However, there are only 24 genes in common. We have explained this now in the figure caption.*

5. Also, for this example you have only highlighted the differences that serve as positive controls. I would like to know what other groups of genes that scSTEM picks out for this example as that will give a better idea of what it will be like to use the tool in a situation where less is known about the outcome.

*Response: Thank you. Indeed, we have noted that other groups of genes could better inform readers about what to expect from scSTEM outputs. Following this comment we have now added the gene names included in all clusters presented in Fig. 5A and Fig. 5B in Additional File 3, as well as all significantly enriched GO terms of these clusters into Additional File 4. As mentioned above, we have also expanded the set of GO terms we show for each cluster.*

In the example for fig 5, the scenario that you consider is essentially: "Find gene clusters and check which ones are overrepresented for NK cells". What I would like to see is a use case which is somewhat more realistic. In many cases, one would need to compare to lots of marker gene lists or GO terms to figure out what is going on. I would like the authors to present a use case where the interpretation and downstream analysis is more challenging and extensive than what is currently the case.

*Response: Thank you for your suggestion. Following this comment we performed additional analysis using scSTEM on a less well annotated subset of cells from spleen tissue [1]. We have added the analysis results to Results section.*

*In the original publication of this data set, the authors did not identify a specific cell type for these cells and so marked this cluster based on its highly expressed genes STC2 and TLX1. We therefore used “STC2_TLX1” to label this cell cluster. We first ran differential expression analysis by Monocle3 to identify the top 3000 differentially expressed genes (DEGs). In UMAP plot, it is observed that the stromal cells are in close proximity to STC2_TLX1 cluster (Additional File 2, Fig. S2). So our following analyses focused on STC2_TLX1 cell cluster and on the stromal cells from the same component. Next, we applied scSTEM to this subset of cells. The trajectory analysis identified 5 paths and we focused on path3 since many STC2_TLX1 cells were mapped to that path (Additional File 2, Fig. S2).*

*scSTEM identifies cluster0 in path3 as significant. This cluster is characterized by a strong decline in expressions, which may signify its role during the cell transition. GO analysis further revealed that cluster0 is likely to associate with “antigen processing and presentation of endogenous antigen” (corrected p-value = 0.004). The discussions in the supplementary materials of human immune cell data set paper [1] suggested this STC2_TLX1 cell cluster may relate to mesenchymal precursors or stem cells. These cells have been reported to activate antigen presentation [2,3]. Although the true cell type of STC2_TLX1 cell cluster remains undetermined, scSTEM analysis may help to annotate this unknown set of cells based on the profiles and function of the clusters.*

6. When two paths are compared as in Fig 5, do you use any multiple hypothesis correction for the tests?

*Response: We note that the number of hypothesis we testes in such comparisons is very small. Specifically, we only compare the overlap of significant cluster groups (same color) in both datasets. Since the number of significant cluster groups in a specific dataset is very small (ranging from 2-5 in the data we tested) the overall number of comparisons is, on average, 10 or so and not many hypotheses are being tested in each comparison. Still, following this comment we revised the software to allow for multiple hypothesis corrections with BH prodecure. Since all the p-values presented in the paper for the comparisons are low (less than 10^-5) such correction did not impact the results we presented.*

7. How easy is it for a future user to modify scSTEM to allow it to use another trajectory inference method?

*Response: While this is not the main focus of the paper, scSTEM is very flexible in terms of which trajectory inference method it can use. Specifically, scSTEM uses Dynverse as the back-end trajectory inference engine and since Dynverse aims to provide a unified representation for all trajectory inference method, any method that would be added to Dynverse will be immediately supported by scSTEM.*

Minor issue:

1 On line 346 there is a reference to 2.2.3 but it is not clear what this corresponds to.

*Response: Thank you for pointing out this. This is a typo. We have changed it to “Summarizing gene expression levels along a path”.*

2 I do not really understand where path 2 with 300 genes comes from in fig 5. Looking at line 4 in the additional_file1 I see a path2 with 301 genes - is this a typo or a misunderstanding from my side? Same with path6 where I find one with 56 genes, but for path7 it seems to be correct with 124 genes.

*Response: Yes, these are typos and we have missed one gene for these sets. The mistake arose from the use of a R program that automatically treated the first row as header and so did not correctly count the number of genes. We have corrected them in the manuscript.*

Reviewer #2:

Summary

In this manuscript, the authors describe a method called scSTEM. This is an adaptation of their previously published STEM method to scRNA seq data. The paper gives a clear overview of the scSTEM algorithm, both the original STEM method and the extensions required for scRNA-Seq. The authors use 3 real datasets to showcase usages of scSTEM. They are quite convincing in demonstrating the usefulness of the method: by providing p-values to cluster assignments, the method simplifies biological interpretation and allows for comparisons between branches. They also provide software on github that is very easy to use and flexible to upstream analysis choices. The shiny app in particular provides a very nice UI that will be useful for non-experienced coders.

scSTEM will be very useful to the community, provided some additions to the paper. In particular, the paper makes several assumptions when extending STEM to scRNA-Seq data which are not properly demonstrated, and it lacks comparisons with appropriate methods to demonstrate superiority.

Major comments

- The authors assert (Line 291-292) that "the changes between milestone nodes is usually linear for genes". However, they do not back this claim by either appropriate reference, or demonstration on real datasets.

*Response: We agree that there is little discussion in the literature about the type of function that should be used to represent genes between milestones (sampling points). We have done extensive work on this topic and have shown that piecewise linear functions are usually good enough *if* the sampling rate is appropriate [4]. We expect that most studies that use scSTEM will attempt to achieve appropriate sampling rates. However, the reviewer is correct that there is no guarantee that the sampling rate is, indeed, appropriate. We have thus explored the expression changes along the edges of trajectory paths (we assume linearity within an edge). Genes expressed in at least 5% of the cells that mapped to the edge were selected for this analysis. For each gene, we used corrected p-values (BH corrections) from F test to evaluate the fit of a linear model. As shown in Additional File 2, Fig. S11, we observe a significant fit for a linear model for over 50% of the genes for internal (non terminal) edges. We observe less agreement for terminal edges but this is in part a function of the way the trajectory is constructed. Terminal edges have much fewer cells and significant than internal edges (since each branching further splits the number of genes) which leads to much noise expression profile that is harder to model using a simple curve. Since the vast majority of cells and genes are assigned to internal edges, the overall linear assumption makes sense for this method. We have also added this into our Discussion section.*

- Each milestone node is mapped to a time point (figure 1 for example) and then each gene is associated with a profile. However, this induces a transformation between pseudotime and the time of the profiles, so that the distance (in pseudotime) between each milestone node becomes the same. While this won't matter at the level of a single path, it might be inappropriate to do so when comparing various paths. Milestone nodes might have very different pseudotimes after branching: this time wrapping may artificially create or remove differences between paths, and the authors need to demonstrate that it is not the case.

*Response: Thank you. We agree that pseudotime along the paths may vary after branching. However, the comparison that scSTEM performs *is not based on the profile* and so does not consider time at all. Specifically, scSTEM compares clusters based on the overlap in the *genes* assigned to significant clusters. It is then up to the user to determine if the two profiles are similar in the two paths (and so, genes in the two sets of cells behave the same way) or different. The reviewer is correct that some of the* *difference can result from differences in timing between the two branches. But this is not something that scSTEM deals with, this is something that users, based on knowledge regarding the specific cell types that are represented by each path can decide. scSTEM only highlights the overlap in terms of genes.*

- No comparisons to other methods are made, especially for 2 things.

+ the common workflow to identify GO terms enriched in a branch (or between branches) is to fit a trajectory, then find DE genes using methods such as those implemented in monocle 2 or 3, GPfates, or tradeSeq

and then use the ranking of p-values as input to GO methods.

+ ImpulseDE (ImpulseDE2) similarly fits profiles to genes (with an impulse model). The authors need to explain how scSTEM differs from this.

*Response: Thank you for your suggestions. As suggested, we have performed benchmark and comparisons with three additional methods. We have added this analysis to our Results section.*

*For DE gene analysis, we have run differential expression analysis using graph_test() function in Monocle 3 and the workflow of DE gene analysis in tradeSeq. GO enrichment was then performed using ranking of p-values. The comparison performed was performed using path 1 (Fig. 2B) from the human fetal immune cell data set and is presented in (Fig. S1B) Since there is no standard metric for comparing gene clustering results, we used NK gene ratio to evaluate the results (number of genes annotated with natural-killer-cell-related functions divided by cluster size or DE gene number). Results indicate that by using clustering and the temporal trajectory scSTEM obtains a higher ratio of NK genes compared to other two methods (5.98% for scSTEM, 3.39% for Monocle 3 and 3.72% for tradeSeq). scSTEM is also more efficient than tradeSeq. Run time for scSTEM on this data was 60s while tradeSeq took more than 10000 seconds on this single path (3000 genes and 4058 cells).*

*For clustering analysis, we have run two comparison methods as well, scHot and tradeSeq on the same path.*

*Results, presented in Fig. S1A show that scSTEM is much faster than the other two methods and produced only 2 significant clusters by grouping similar profiles (tradeSeq identified 66 clusters which is likely too many to analyze). We next compared GO enrichment for the top five larges clusters for both methods. Go analysis has identified the term “regulation of natural killer cell mediated cytotoxicity” in scSTEM clusters (corrected p-value = 0.018, Fig. S1A) while the other two methods failed to identify any NK related GO terms for the top five clusters. Additionally, a user would have to screen for all 66 clusters to identify cluster of interest and tradeSeq is also much more computationally expensive than scSTEM. As for scHOT, it does not scale to the number of cells and genes in this dataset. Specifically, scHOT clusters genes by computing pairwise spearman correlation followed by hierarchical clustering. Such operations are very computationally expensive for large dataset and would take days for the entire datasets we analyzed. We thus only applied scHOT to the top 300 marker genes in the NK cell path (as in Fig. 2A). We compared the results to scSTEM using GO functional enrichment as the criteria. See Fig.* S1A for the *evaluation results. As can be seen, unlike scSTEM, scHOT was not able to identify NK cell related GO terms as enriched in any of the gene clusters it identified.*

*For two other method the reviewer mentions we were unable to obtain results. We did not find clear documentations for GPfates in the GitHub repo (https://github.com/Teichlab/GPfates) and thus GPfates was not used in comparison. As for ImpulseDE2, it produced the following error message when we tried to run it:*

*Error in estimateSizeFactorsForMatrix(counts(object), locfunc = locfunc, :*

*every gene contains at lest one zero, cannot compute log geometric means*

- While the paper is very strong in terms of real data, it is lacking simulations. The dynverse, already used by the authors, provides a suitable simulation framework.

*Response: Thank you for your suggestion. The dynverse package indeed has provided dyngen and dyntoy for creating simulated trajectories. These simulators can create desirable trajectory topology by simulating proper gene network structure that generates the certain topology structure and by simulating gene expressions with Poisson count models. However, these simulators are primarily designed to simulate gene expressions and regulatory dynamics related to certain trajectory topology structures. For gene clustering, the true cluster structures are yet not strictly defined, or they are not as obvious as trajectory topology structures which can often be visualized by dimensional reduction (e.g. for trajectory simulation, the cell organizations are clear for topology structure such as linear trajectory, bifurcation or trifurcation). A good “ground truth” for a simulator to follow remains difficult to be defined.*

*Additionally, unlike benchmarking trajectory inference, which has a well-defined underlying truth. The clustering methods we tested in our manuscript explore different properties of expression data. For example, scSTEM and tradeSeq try to identify genes with similar expression patterns, while scHOT identifies genes with high level of correlations. Creating any simulation based on a certain property in may possibly be biased towards a certain clustering method. In our analysis, we used functional annotation as a metric to evaluate clustering methods in a relatively unbiased way. However, different clustering method still has its own advantages.*

Minor comments

- Filtering, as reported by the authors, can have a large impact on clustering results, although scSTEM might be less impacted. Some sensitivity analyses and comparison with usual clustering methods would help show this.

*Response: Thank you for your suggestion. Following this comment, In our revised manuscript we performed additional analysis which used different numbers of input genes to scSTEM to test its robustness to filtering (See Additional File 2, Fig S10). Briefly, we ranked the top differentially expressed cell-type marker genes for human immune cell data set by their corrected p-values (corrected p-value < 0.05 and fold change > 1.5, see also Methods section). Based on this selection and ranking we ran scSTEM with various size of input genes (1000, 2000, 3000, 4000, 5000, 6000).*

*To compare the cluster assignments across different input sizes, we extracted the 127 genes found in significant clusters using the first input setting (1000 genes) and tested if they remained in the same class when using more genes. Similarity of cluster assignments was evaluated by adjusted rand index (ARI). The comparison results show that cluster assignments remain relatively stable for sizes up to 5000 genes which is usually above the number most users select. We performs permutation analysis that further supported this observation. Based on these results we recommend that users filter genes prior to clustering by using the STEM provided option or using other methods such as Monocle3 is used (Fig S10B).*

- Related, in Line 183, the authors perform a hypergeometric test using all genes as background. This is incorrect since none-filtered genes cannot be in the clusters. The background should be all genes that pass filtering.

*Response: We disagree. The analysis performed by scSTEM involves two steps. The first is filtering non changing genes and the second is clustering the ones that are retained. There is no reason to not use the entire set of genes to compute the final outcome since its based on the two steps and not just on the clustering. Its true that when performing comparison to other methods we should use the same background (all genes) which is what we did, but we do not agree that we cannot use the entire set of genes as background for enrichment analysis. In fact, STEM, which has been used thousands of times in publications uses the same background and to date this has been widely accepted.*

- It would be interesting to show how some genes fit to their assigned profile, for a few clusters, as a supplementary figure.

*Response: Thank you for your suggestion. We have added detailed gene profile figures of path 2 clusters (Fig. 5A and Fig. 5B) from human fetal immune cell data set into Additional File 2, Fig. S3.*

- Some case studies have too many lineages, especially with monocle 3 which tend to find lots of small lineages that are data artifacts. Tuning the parameters will remove those and produce a more likely trajectory.

*Response: We agreed that the minimum spanning tree-based inference method, such as monocle 3 would create some small, trivial branches. We have added relevant discussions into the Discussion section to inform our readers of this situation. We have also added parameters panel for monocle3 and PAGA to let the readers tune the parameters until they have a satisfying trajectory result.*

- Can the comparison between branches be extended to more than two paths?

*Response: Thank you for your suggestion. This would potentially be an interesting question to investigate in the future. While we do not have a good idea on how to do this, we now mention this as an option for future work in Discussion.*

- Are the authors planning to submit the package to CRAN or Bioconductor? This would increase its diffusion (with the understanding that package with shiny apps are hard to register)

*Response: Thank you. We are planning to submit the package to CRAN/Bioconductor in the future. Currently scSTEM depends on packages on CRAN, Bioconductor, GitHub and external containers such as singularity or docker. Therefore, the dependency environment would also be challenging to be handled if package is submitted to CRAN or Bioconductor. We will be working on this to find a good solution.*

- The author did provide the scSTEM package and the data but failed to provide the code needed to reproduce the paper.

*Response: We have uploaded all three sample data sets to GitHub repo. Users may use the scSTEM GUI to reproduce the results in the paper. Further, we have uploaded code that reproduces results of the supplementary figures and GO plots in the main figures to the scSTEM GitHub repository (https://github.com/alexQiSong/scSTEM, DOI:10.5281/zenodo.6331254). Users may find analysis code and data under the folder /analysis to reproduce results in this research.*

*References*

*1. Cao J, O’Day DR, Pliner HA, Kingsley PD, Deng M, Daza RM, et al. A human cell atlas of fetal gene expression. Science (80- ). 2020;*

*2. Chan JL, Harrison JS, Ponzio NM, Rameshwar P. Mesenchymal Stem Cells (MSC) Exhibit Antigen Presenting (APC) and Phagocytic Properties: Implicatins to Bnone Marrow Failure during Inflammation. Blood. 2004;*

*3. Chan JL, Tang KC, Patel AP, Bonilla LM, Pierobon N, Ponzio NM, et al. Antigen-presenting property of mesenchymal stem cells occurs during a narrow window at low levels of interferon-γ. Blood. 2006;*

*4. Sefer E, Kleyman M, Bar-Joseph Z. Tradeoffs between Dense and Replicate Sampling Strategies for High-Throughput Time Series Experiments. Cell Syst. 2016;*

**Second round of review**

**Reviewer 1**

The authors have addressed all my comments regarding the main text. The documentation on the GitHub page seems to have improved and I was able to successfully install it this time around.

**Reviewer 2**

We thank the authors for their answers to our questions and the work they provided. 3 out 4 major comments and all minor comments but 1 have been answered to our satisfaction, and we believe the authors will be able to correctly answer our remaining questions:

Major comments:

1/ This clarification on linear fit is very interesting and convincing. Providing this task as a function of the scSTEM package would allow curious users to run this sanity check on their own data. If that task is too computationally intensive, it could maybe be run on a random subset of genes.

2/ This is now much clearer for me. Adding this as part of the discussion would also be informative for readers.

3/ No further questions.

4/ We agree that dynverse is not yet suited for a benchmark of clustering. However, scSTEM goes beyond clustering to identify significant clusters. It would be interesting to see how those genes selected in the clusters overlap with the DE genes on a dynverse simulation. Showing both what is detected in scSTEM and what is not detected will give users a better sense of how to interpret the output of the method.

Minor comment.

Regarding the hypergeometric test: the authors state that their method is filtering + clustering. However, that is entirely absent from the figure 1 which presents their method. Moreover, filtering is not unique to scSTEM. It is therefore not possible to know if the enrichment comes from the filtering step or the scSTEM method. What needs to be demonstrated is the added enrichment that scSTEM procures, and using all genes as background obscures this.

All other minor comments have been addressed.

**Authors Response**

**Point-by-point responses to the reviewers’ comments:**

Reviewer #2

We thank the authors for their answers to our questions and the work they provided. 3 out 4 major comments and all minor comments but 1 have been answered to our satisfaction, and we believe the authors will be able to correctly answer our remaining questions:

Major comments:

1/ This clarification on linear fit is very interesting and convincing. Providing this task as a function of the scSTEM package would allow curious users to run this sanity check on their own data. If that task is too computationally intensive, it could maybe be run on a random subset of genes.

*Response: As suggested we have added this functionality into our GUI. It can now be run as an option in the panel labeled “Step 4. Check paths”. With this, users can conveniently generate plot that shows linear fit for a trajectory path.*

2/ This is now much clearer for me. Adding this as part of the discussion would also be informative for readers.

*Response: We have added this to Discussion as suggested.*

3/ No further questions.

4/ We agree that dynverse is not yet suited for a benchmark of clustering. However, scSTEM goes beyond clustering to identify significant clusters. It would be interesting to see how those genes selected in the clusters overlap with the DE genes on a dynverse simulation. Showing both what is detected in scSTEM and what is not detected will give users a better sense of how to interpret the output of the method.

*Response: As suggested, we now performed this analysis. For this we simulated scRNA-seq data using the dyntoy tool from dynverse toolkit. Dyntoy is able to simulate scRNA-seq data with prespecified trajectory structure and DEGs. We simulated scRNA datasets for a number of different trajectories including linear, bifurcating, and multifurcating, each using 10000 cells and 3000 genes. For each topology structure, we generated three datasets with 10%, 30%, and 50% genes as DEGs. Note that given the way Dyntoy works, not all of these genes are changing over time. Some can be DE in specific time points but not others. The details of simulation are described in Additional file 2 (subsections titled Generation of synthetic scRNA-seq datasets and Evaluation of scSTEM using synthetic scRNA-seq data). The results, presented in Additional file 2: Fig S12, indicate that *all* genes in scSTEM-identified clusters were indeed from the simulated DEGs, though several simulated DE genes were not part of the significant clusters, this is a function of the way the DE genes are generated and not related to the ability of scSTEM to infer significant trajectories. To show that this is indeed the case we further partitioned the DEGs into two sets: 1) common genes, defined as those DE genes assigned to significant scSTEM clusters and 2) DEG-specifc genes, which are DEGs not identified by scSTEM. We computed absolute values of Pearson correlation coefficients between the expression of each gene set and their corresponding cell pseudotime values. Results, presented in Additional file2: Fig. S12 show that while common genes exhibit high correlation with cell pseudotime values, DE genes not assigned to scSTEM clusters are much less correlated with the cell pseudotime values. Thus, scSTEM can correctly identify all clusters composed of temporally changing genes, suggesting that DEGs identified by scSTEM include stronger biological signals than other DEGs.*

Minor comment.

Regarding the hypergeometric test: the authors state that their method is filtering + clustering. However, that is entirely absent from the figure 1 which presents their method. Moreover, filtering is not unique to scSTEM. It is therefore not possible to know if the enrichment comes from the filtering step or the scSTEM method. What needs to be demonstrated is the added enrichment that scSTEM procures, and using all genes as background obscures this.

*Response: Thank you for your comment. To clarify, our analysis involves two steps, 1) filtering genes without using the original STEM criteria and using this filtered count matrix as input to scSTEM; 2) scSTEM analysis. This step includes filtering genes by checking number of missing values and log fold change at each time point, as described in the original STEM paper. In the original paper we argued (and this has been adopted very widely since) that both filtering steps should be considered as part of the method and evaluation when performing enrichment analysis. The reason is that the filtering is based on observed expression data for genes and that is definitely different between different datastes. It’s true that this is not a unique contribution of scSTEM, but users of the tool will still rely on these p-values which are a function of applying both methods. Still, to test the advantage of using scSTEM after filtering we re-performed hypergeometric test using genes that passed step1 filtering as the background (3000 genes). When using this set the new p-value for enrichment of NK cell markers is still significant for one cluster (p-value = 0.012 for C0P2, cluster 0 in path 2)) though not significant for the other (p-value = 0.240 for C2P7, cluster 2 in path 7)). We have added this to Results.*
